# Genome-Wide Analysis of the DREB Subfamily in *Saccharum spontaneum* Reveals Their Functional Divergence During Cold and Drought Stresses

**DOI:** 10.3389/fgene.2019.01326

**Published:** 2020-02-05

**Authors:** Xing Huang, Xiupeng Song, Rongfa Chen, Baoqing Zhang, Changning Li, Yongsheng Liang, Lihang Qiu, Yegeng Fan, Zhongfeng Zhou, Huiwen Zhou, Prakash Lakshmanan, Yangrui Li, Jianming Wu

**Affiliations:** ^1^Sugarcane Research Institute, Guangxi Academy of Agricultural Sciences, Guangxi Key Laboratory of Sugarcane Genetic Improvement, Ministry of Agriculture Key Laboratory of Sugarcane Biotechnology and Genetic Improvement (Guangxi), Nanning, China; ^2^Nanning Institute of Agricultural Sciences, Guangxi Academy of Agricultural Science, Nanning, China

**Keywords:** DREB, transcription factor, phylogenetic tree, abiotic stress response, sugarcane

## Abstract

Drought and cold stresses are the main environmental factors that affect the yield of sugarcane, and *DREB* genes play very important roles in tolerance to drought, cold, and other environmental stresses. In this study, bioinformatics analysis was performed to characterize *Saccharum spontaneum SsDREB* genes. RNA sequencing (RNA-seq) was used to detect the expression profiles of *SsDREBs* induced by cold and drought stresses. According to our results, there are 110 SsDREB subfamily proteins in *S. spontaneum*, which can be classified into six groups; 106 of these genes are distributed among 29 chromosomes. Inter- and intraspecies synteny analyses suggested that all DREB groups have undergone gene duplication, highlighting the polyploid events that played an important role in the expansion of the DREB subfamily. Furthermore, RNA-seq results showed that 45 *SsDREBs* were up- or downregulated under cold stress; 35 of them were found to be involved in responding to drought stress. According to protein–protein interaction analysis, SsDREB100, SsDREB102, and SsDREB105 play key roles during the response to cold stress. These results reveal that functional divergence exists between collinear homologous genes or among common origin genes in the DREB subfamily of *S. spontaneum*. This study presents a comprehensive analysis and systematic understanding of the precise mechanism of *SsDREBs* in response to abiotic stress and will lead to improvements in sugarcane.

## Introduction

DREB (dehydration-responsive element-binding protein) is a type of plant-specific transcription factor that can specifically bind to DRE/CRT elements in the response to abiotic stresses such as drought and low temperature (Gilmour et al., 1998; Liu et al., 1998). DREB belongs to the AP2 superfamily and contains one highly conserved AP2/ERF domain. Seven key amino acids in the AP2 domain play an important role in CRT/DRE element binding, namely, four R residues, two W residues, and one V residue (Chen et al., 2007). Among them, the V^14th^ is the key site of interaction (Cao et al., 2001) and is a characteristic of the subfamily. The DREB subfamily can be divided into six groups (A1–A6) (Sakuma et al., 2002; Liu et al., 2017b) or four clades (I, II, III, and IV) (Lakhwani et al., 2016; Wang et al., 2019) according to the structural characteristics of the protein sequence.

The function of DREB was first identified in *Arabidopsis*. AtDREB1 can improve the tolerance of *Arabidopsis* to drought, and AtDREB2 can increase the survival rate of *Arabidopsis* under cold stress (Liu et al., 1998). The factors have since been isolated and characterized in many plant species, showing that DREBs are involved in a variety of stress responses (Peng et al., 2011; Movahedi et al., 2012; Sun et al., 2013; Wang et al., 2013; Li et al., 2018; Jangale et al., 2019).

Previous reports have identified DREB1/CBF as key transcription factors that function in cold acclimation. CRPK-14-3-3s-CBF and CRLK-MEKK-MPK-ICE-CBF comprise the main signal transduction cascades (Shi et al., 2018b). C-repeat-binding factor (CBF) proteins recognize and bind to the conserved CRT/DRE *cis*-element (CCGAC) in the promoters of a subset of COR (cold-regulated) genes, inducing expression and enhancing freezing tolerance in plants (Yang et al., 2011; Movahedi et al., 2012; Guo et al., 2018; Shi et al., 2018b). MbDREB1, isolated from *Malus baccata*, can activate expression of *COR15* and *rd29B via* ABA-independent and ABA-dependent pathways (Yang et al., 2011). Constitutive expression of OsDREB1 in *Arabidopsis* results in a higher tolerance to drought and freezing stresses (Dubouzet et al., 2003; Ito et al., 2006). *ARAG1*, a *DREB* gene upregulated by ABA or drought treatment in rice, plays a role in drought tolerance and seed germination (Zhao et al., 2010). *GmDREB2*, a member of the A-5 group of the DREB subfamily, is induced by drought, low temperature, and ABA, and overexpression of *GmDREB2* results in enhanced tolerance to drought without growth retardation (Chen et al., 2007). Ten ScDREB proteins have been isolated from *Syntrichia caninervis*, a typical desiccation-tolerant moss, exhibiting differential expression under ABA treatment and cold and other stresses. Among them, ScDREB3 and ScDREB5 enhanced cold tolerance in yeast, and ScDREB8 and ScDREB10 conferred tolerance to osmotic, salt, cold, and high-temperature stresses (Li et al., 2016b).

Although transgenic tomatoes do not exhibit an increase in freezing tolerance when overexpressing *LeCBF1* or *AtCBF3*, the *LeCBF1* gene has been found to be cold inducible (Zhang et al., 2004). *ZmDREB4.1* belongs to the A-4 group and is not induced by biotic or abiotic treatment, but it does play an important role in the negative regulation of plant growth and development (Li et al., 2018). In brief, there are great differences in function in response to abiotic stress for the different *DREB* genes in different species, even though they all have a common origin. Therefore, genome-wide and systematic analysis of DREB family members is essential to discern their mechanisms in abiotic stress tolerance.

The amounts and distribution of DREBs in different plants vary greatly. Whole-genome duplication (WGD) is thought to have played important roles in the expansion of the *DREB* gene family, especially for plants that have undergone recent WGD events, such as τ for Commelinid-specific WGD, or family-level WGD events α/β for Brassicaceae and ρ for Poaceae (Wang et al., 2019). Expansion of the DREB subfamily has allowed for more detailed and complex functional differentiation or redundancy. For instance, 79 members have been identified in zoysia grass, with seven being downregulated and 12 and four members highly expressed after 2 h and 3 days of cold stress, respectively (Zhao et al., 2018). There are 39 *DREB* genes in the jujube genome, and ZjDREB39 has been confirmed to positively participate in jujube fruit ripening (Zhang and Li, 2018). Thirty DREB proteins of *Vigna radiata*, a valuable legume crop for which drought is one of the major factors hindering its growth, have been characterized, five of which are highly expressed when the plant has been exposed to drought stress by withholding water for 7 days (Labbo et al., 2018). There are 81 and 99 DREB-encoding genes in *Musa acuminata* (A genome) and *Musa balbisiana* (B genome), respectively, twice as many in *Citrus sativus* and more than 1.5 times that in *Arabidopsis* (Lakhwani et al., 2016). Only 41 DREB proteins have been identified in barley (*Hordeum vulgare*); most of them exhibit low expression during development, but for many of them, expression is strongly induced under salinity, dehydration, and ABA treatments (Guo et al., 2016). Studies on 30 *DREB* genes of *Morus notabilis* suggest different response models between leaves and roots for the same gene (Liu et al., 2015). Therefore, comprehensive analysis of DREBs is essential to precisely understand the mechanism of abiotic stress tolerance.

Sugarcane is a C4 plant with a high photosynthetic rate and ability to accumulate biomass. As the main sugar crop in the world, sugarcane is planted in tropical and subtropical regions (Su et al., 2014; Pang et al., 2015; Xu et al., 2018). Therefore, low temperature, frost, drought, and water shortage often cause great economic losses to sugarcane production and are the primary environmental factors limiting production of this plant. Two *DREB2* genes from wild sweet cane, *Saccharum spontaneum*, have been cloned and characterized (Yao et al., 2016). cDNA-SCoT revealed 120 differentially expressed genes in *S. spontaneum* under drought stress (Wu et al., 2018b), and after stopping water for 4, 7, and 11 days, 1,325 significantly differentially expressed genes (DEGs) were obtained by RNA sequencing (RNA-seq) (Wu et al., 2018a). In addition, expressions of *DREB1/CBF*, *WRKY*, and *bHLH* as well as other TF genes are induced in *S. spontaneum* when exposed to low-temperature conditions (Selvarajan et al., 2018). Furthermore, 2,583 and 3,302 genes in *S. spontaneum* were reported to be up- and downregulated, respectively, during cold stress, including a *DREB2* gene with eightfold upregulation (Dharshini et al., 2016).

Cultivated sugarcane is usually a polyploid interspecific hybrid, combining high sugar content strain (*Saccharum officinarum*) with a strain with disease resistance and ratooning (*S. spontaneum*) (Zhang et al., 2018), but a shortage of genomic information hinders exploration of tolerance to abiotic stress and impedes breeding improvement. In 2018, the genome sequence of an *S. spontaneum* haploid was assembled to 3.13 Gb, with anchoring on 32 chromosomes (Zhang et al., 2018), facilitating and accelerating sugarcane research and breeding. Because DREB proteins play a very important role in tolerance to abiotic stress, our study of *DREB* genes will provide information for understanding their role in the tolerance of *S. spontaneum* to cold and drought stresses and will provide a foundation for utilizing wild germplasm resources to improve the drought resistance of sugarcane.

## Materials and Methods

### Identification and Characterization of DREB Proteins in *S. spontaneum*

To identify *S. spontaneum* DREB proteins, DREB and AP2/ERERBP were used for *S. spontaneum* genome annotation result searches (Zhang et al., 2018) (the genome assembly and gene annotation are available through accession number QVOL00000000, BioProject number PRJNA483885, and BioSample number SAMN09753102 in the NCBI database). Next, all protein sequences in the genome were searched against the conserved AP2 domain (pfam ID: PF00847, http://pfam.xfam.org/family/PF00847) using HMMER software (v3.2.1) (Wheeler and Eddy, 2013). After preliminary identification results were obtained from the above two methods, a reliable domain protein sequence was established to construct a species-specific hidden Markov sequence file, with an *e*-value <0.001 indicating a candidate family gene. Three online tools of the domain identification website were then used to detect whether the AP2 domain exists, including NCBI CDD (https://www.ncbi.nlm.nih.gov/Structure/bwrpsb/bwrpsb.cgi), SMART (http://smart.embl-heidelberg.de/), and PFAM (http://pfam.xfam.org/). To identify SsDREB proteins, alignment of the AP2 domain for each filtered gene was performed using MEGA X (Kumar et al., 2018) (https://www.megasoftware.net/), and all of the candidate genes with a V (valine) at the 14th amino acid position and an E (glutamic acid) at the 19th position in the AP2 domain were selected as SsDREB proteins. Ultimately, 110 SsDREB proteins were identified.

For all 110 SsDREB proteins, the compute p*I*/MW tool of the ExPASy server (http://www.expasy.org) (Artimo et al., 2012) was used to calculate protein features, such as the molecular weight (MW) and theoretical isoelectric point (*p*I). Exon–intron structures of the *SsDREB* genes were examined by comparing CDS sequences and the corresponding genomic sequences using online Gene Structure Display Server (GSDS 2.0: http://gsds.cbi.pku.edu.cn/) (Hu et al., 2015) and were checked using TBtools (https://github.com/CJ-Chen/TBtools). The MEME motif searching tool (Bailey et al., 2009) (http://meme-suite.org/) was employed to identify motifs within the SsDREB subfamily, with the following parameters: any number of repetitions, maximum of 10 mismatches, and an optimum motif size of 6–70 amino acid residues.

### Phylogenetic Analysis of SsDREBs

SsDREB and AtDREB protein sequences were aligned using the Clustal W program and employed for phylogenetic analysis using MEGA X; unrooted phylogenetic trees were constructed by the neighbor joining (NJ) method based on the JTT amino acid substitution model. To deduce the evolutionary relationship of DREB proteins between *S. spontaneum* and other plants, 317 protein sequences from six other species (*Amborella trichopoda*, *Arabidopsis thaliana*, *Sorghum bicolor*, *Oryza sativa* subsp. *japonica*, *Setaria italica*, and *Zea mays*) were aligned and used to construct the phylogenetic tree. To compare the composition of the DREB subfamily, additional DREB proteins were downloaded from Plant Transcription Factor Database (PlantTFDB 4.0: http://planttfdb.cbi.pku.edu.cn/index.php) (Jin et al., 2017). Evolutionary analyses were conducted in MEGA X, and the number of bootstrap replicates was 1,000. The species and sequences are shown in **Supplementary Table 2**.

### Chromosomal Locations and Synteny Analysis

The chromosomal locations of the *SsDREB* genes were extracted from the *S. spontaneum* genome annotation gff3 file for synteny analysis. Analyses of duplication events and inter- and intraspecies synteny were investigated using MCScanX (Multiple collinear scanning toolkits) (Wang et al., 2012). Chromosome location images and synteny analysis images were then produced by TBtools software. Additionally, TBtools software was used to calculate the synonymous (Ks) and non-synonymous (Ka) substitution rates of tandem duplicated *SsDREB* gene pairs.

### Identification of *cis-*Acting Regulatory Elements in *SsDREB* Promoters

The sequence (2 kb) upstream of the transcriptional start site was used to identify *cis*-elements in the promoter sequences of *DREB* family genes in *S. spontaneum*. To evaluate whether other genes were present in the sequence, these sequences were first blasted in NCBI and in the *S. spontaneum* genome. The filtered sequences were then submitted to PlantCARE (http://bioinformatics.psb.ugent.be/webtools/plantcare/html) to identify *cis*-acting regulatory elements (Lescot et al., 2002).

### Determination of the Transcriptional Level of the *SsDREB* Genes Responding to Cold and Drought Stresses

*S. spontaneum* GX87-16 was cultivated in an artificial climatic chamber at 28°C and a 14/10-h photoperiod (day/night). One plant was planted in each pot with a total of 50 pots. For cold stress, 24 pots were placed in a low-temperature incubator (4°C) for 3 or 6 days. After 3 days of low-temperature culture, the plants in six pots were collected; the plants in the other six pots were collected after 3 days of recovery culture under normal conditions. The same operation was performed for the plants treated at low temperature for 6 days.

For drought stress, irrigation of 12 plants was stopped, and the relative water content of the soil was determined by drying and weighing. After stopping water for 8 days (the soil absolute water content for drought stress was 7–8%) and reaching a moderate drought level, the plants in six pots were collected; the plants in the other six pots were collected after 3 days of recovery culture under normal conditions. All treatments were sampled with three biological repeats, including the control for each treatment. All of the sampled leaves were cut into segments, the midrib was removed, and the material was frozen in liquid nitrogen and stored at −80°C.

Total RNA was isolated using TRIzol reagent according to the manufacturer’s instructions (Invitrogen, Carlsbad, CA, USA). The RNA integrity was assessed using RNA Nano 6000 Assay Kit of the Agilent Bioanalyzer 2100 system (Agilent Technologies, CA, USA). Equal amounts of total RNA for every sample were used to construct an RNA-seq library. Sequencing libraries were generated using NEBNext^®^ Ultra™ RNA Library Prep Kit for Illumina^®^ (NEB, USA) following the manufacturer’s instructions, and index codes were added to attribute sequences to each sample. The library quality was assessed using an Agilent Bioanalyzer 2100 system (Agilent Technologies, CA, USA).

The high-quality library preparations were sequenced with the Illumina GAII platform, and a total of 14,360,876,831-bp paired-end reads data were generated. All of the data can be accessed in SRA under ID PRJNA549834. The obtained reads were used for blast searches against the coding sequences of *SsDREB* genes. A total of 92 *SsDREB* genes were filtered and used in differential expression analysis. FPKM (fragments per kilobase of transcript per million fragments mapped) values were used to quantify transcript expression (Trapnell et al., 2010), as calculated based on the mapped transcript fragments, transcript length, and sequencing depth. Differential expression analysis was performed using the DEGseq R package (Wang et al., 2010). *P* values were adjusted using *q* values, and *p* < 0.05 and |log_2_ fold change| >1 were set as the threshold for significantly differential expression.

All genes were annotated for protein function using InterProScan (http://www.ebi.ac.uk/interpro/) and BLASTX against the NCBI nr database. The resulting InterPro and BLAST annotations were converted into Gene Ontology (GO) annotations, and all GO terms were mapped to GO categories. The statistical significance of the functional GO enrichment was evaluated using Fisher’s exact test within Blast2GO [false discovery rate (FDR) < 0.05] (Conesa et al., 2005). Significantly enriched Kyoto Encyclopedia of Genes and Genomes (KEGG) pathways were identified with KOBAS 2.0 (Xie et al., 2011) using a hypergeometric test and the Benjamini–Hochberg FDR correction. In addition, CORNET 2.0 (De Bodt et al., 2012) was applied to assess co-expression and protein–protein interaction (PPI) data for the DEGs.

## Results

### Identification of *SsDREB* Genes and Structure Analysis

**Figure 1:**
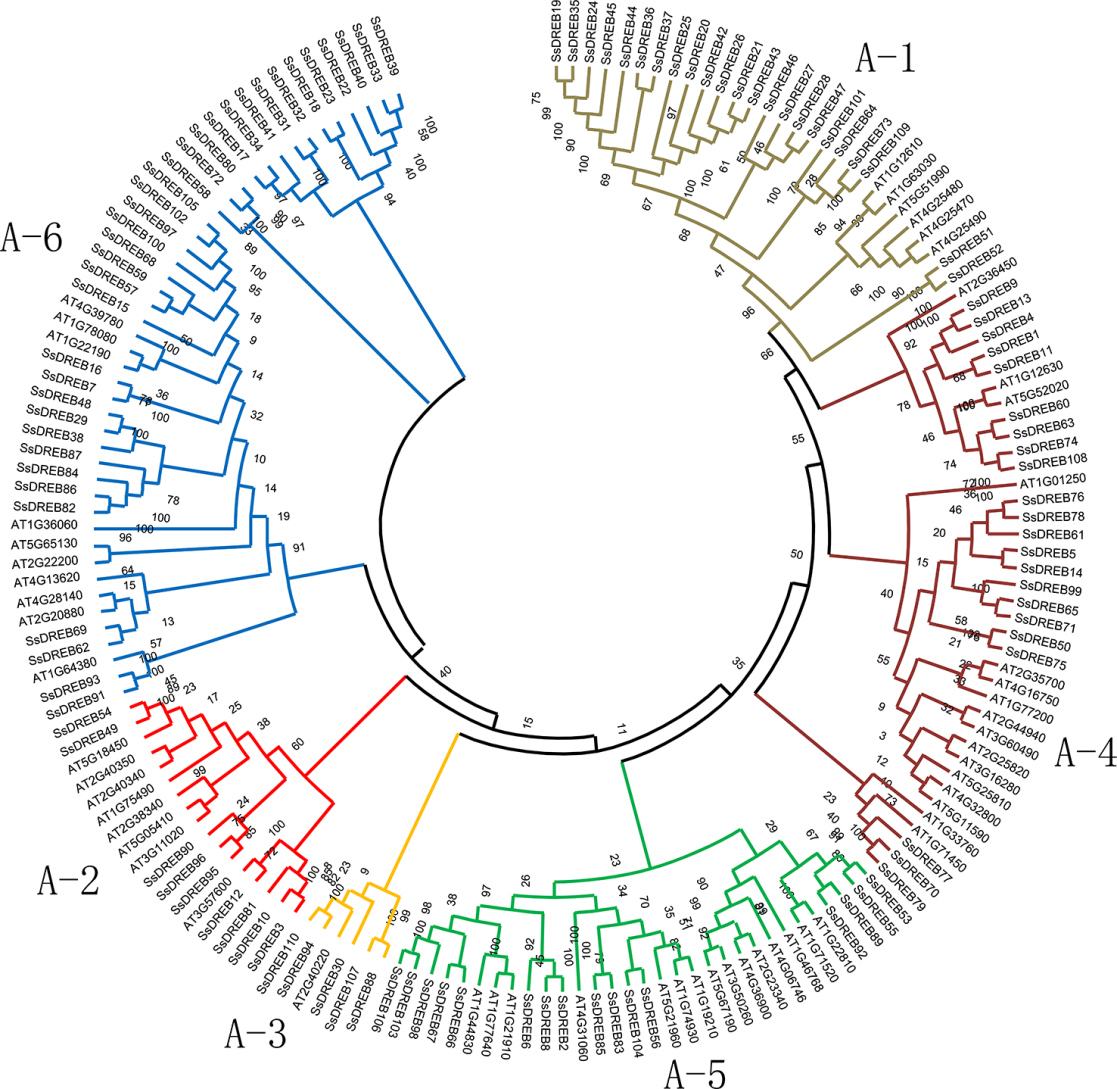
Phylogenetic analysis of DREB proteins between *S. spontaneum* and *Arabidopsis*. A total of 165 conserved protein domains were aligned using Clustal W, and the phylogenetic tree was constructed by MEGA X using the neighbor joining method with 1,000 bootstrap replicates. The sequences of *SsDREB* and *AtDREB* can be found in **Supplemental Table 2**. The branches of the six groups are marked with different colors.

**Figure 2:**
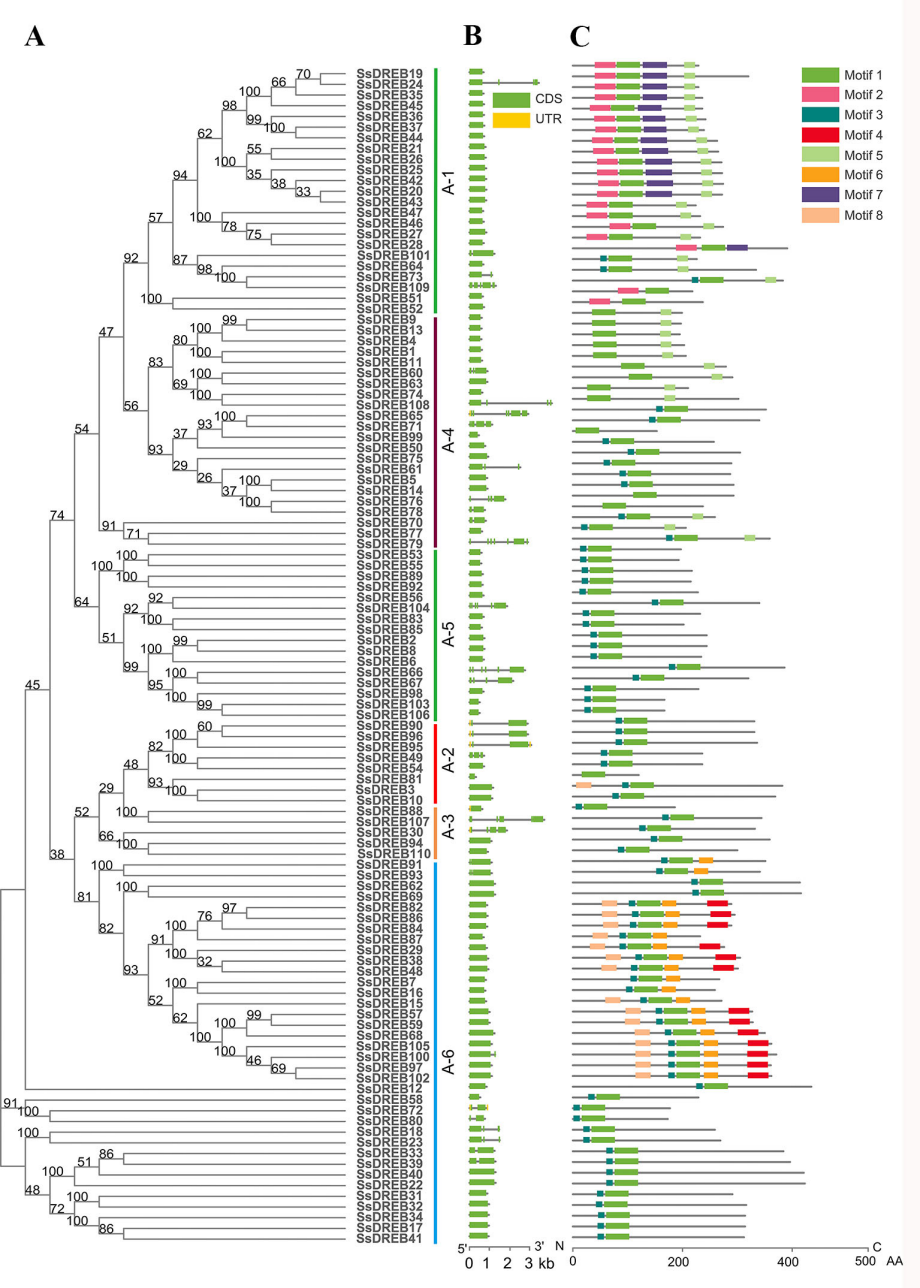
Analysis of phylogenetic relationships, gene structures, and protein motifs and domains of DREBs in *S. spontaneum*. **(A)** Phylogenetic tree of SsDREBs. Alignments of the 110 DREB AP2 domains in *S. spontaneum* were used to construct the tree using the neighbor joining method with MEGA X. The 110 SsDREB proteins clustered into six groups (*A-1* to *A-6*). **(B)** The exon/intron structure of each *SsDREB* gene is proportionally displayed in terms of the scale at the *bottom*. *Green boxes* represent exons, *gray lines* represent introns, and *yellow boxes* represent untranslated regions. **(C)** Conserved motif analysis of SsDREB proteins using MEME tools (Multiple Expectation Maximization for Motif Elicitation, http://meme-suite.org/tools/meme). The order of the motifs is consistent with their position in the protein sequence. *Different colored boxes* represent different conserved motifs.

The AP2 domain was used to search in the published *S. spontaneum* genome database, and genome annotation lists were searched for DREBs. All of the candidate SsDREB proteins were aligned with that of *Arabidopsis*, and a total of 110 *DREBs* were identified in the *S. spontaneum* genome, which were named *SsDREB1* to *SsDREB110* (**Figures 1** and **2A**). Their sequence information and chromosome locations are listed in **Supplementary Table 1**. Detailed analysis of the SsDREB proteins showed that the length of the amino acid sequence varied from 120 aa (SsDREB81) to 614 aa (SsDREB97). The predicted MWs and *p*Is vary from 12.80 to 64.93 kDa and from 4.22 (SsDREB99) to 11.27 (SsDREB6), respectively. The gene structures of *SsDREBs* were investigated by alignment of cDNA sequences and corresponding gDNA sequences. Thirty-two *SsDREB* genes have introns, with exon numbers ranging from 2 to 7 (**Figure 2**). A total of eight conserved motifs were found in the SsDREB proteins using MEME software (**Figure 2**). In addition to the AP2 domain (motif 1) and the long nuclear localization specific sequence (NLS, motif 2), a short NLS and an activated domain rich in aspartic acid and glutamic acid were noted as motifs 3 and 4, respectively (**Supplementary Figure S1**).

### Phylogenetic and Statistical Analysis of Groups of *SsDREB* Genes

To explore the phylogenetic relationship of SsDREB proteins in *S. spontaneum*, two phylogenetic trees were constructed using the neighbor-joining method based on multiple sequence alignments of the AP2 domain in DREB (**Supplementary Table 2**). According to the phylogenetic results, SsDREB proteins can be divided into six groups, corresponding to groups A-1 to A-6 in *Arabidopsis* (**Figure 1**). The largest group is A-6, with 35 SsDREB members, followed by group A-1 with 23. The smallest group is A-3, with only five members.

To better understand the composition of the DREB subfamily in *S. spontaneum*, the AP2 domain of 876 DREB proteins identified from 16 other plants (**Table 1**) and 317 DREBs of six species (four belonging to Poaceae for monocots; *A. trichopoda* is a basal angiosperm and *Arabidopsis* is a model dicotyledon plant) were used to construct the phylogenetic tree (**Supplementary Figure S2** and **Supplementary Table 2**). The comparison revealed that the number of DREB proteins in *S. spontaneum* was far greater than that of the other species, with nearly five times as many as in *A. trichopoda* and twice as many as in sorghum and *Arabidopsis*. This result suggests that the DREB subfamily of *S. spontaneum* has undergone significant expansion. In addition, there were considerable differences in the degree of expansion among the different groups. Compared with *A. trichopoda*, the DREBs have expanded three (A-2) to nine (A-6) times in *S. spontaneum*.

**Table 1:** Statistics of DREB subfamily in selected 17 species.

Species name	Number of DREB proteins in every group						Total	Genome size (Mb)
	**A-1**	**A-2**	**A-3**	**A-4**	**A-5**	**A-6**		
*Amborella trichopoda*	3	3	1	8	4	4	23	748.00
*Arabidopsis thaliana*	6	8	1	16	15	9	55	125.00
*Saccharum spontaneum*	23	9	5	22	16	35	110	3,130.00
*Sorghum bicolor*	10	5	2	17	9	8	51	730.00
*Arabidopsis lyrata*	4	7	1	20	15	10	57	206.7
*Brachypodium distachyon*	18	4	0	15	8	9	54	260.00
*Fragaria vesca*	2	6	1	13	6	5	33	240.00
*Musa acuminata*	8	7	3	27	22	15	82	472.21
*Oryza sativa subsp. japonica*	10	9	1	13	14	9	56	466.00
*Physcomitrella patens*	0	5	2	22	13	2	44	480.00
*Prunus persica*	7	6	1	15	7	7	43	224.60
*Selaginella moellendorffii*	0	4	1	6	2	3	16	212.00
*Setaria italica*	4	7	12	17	15	10	65	510.00
*Thellungiella parvula*	4	7	1	18	14	11	55	140.00
*Utricularia gibba*	3	3	1	14	14	7	42	82.00
*Vitis vinifera*	5	2	0	8	3	5	23	490.00
*Zea mays*	10	9	1	18	15	14	67	2,300.00

### Chromosomal Location and Synteny Analysis of *SsDREB*s

**Figure 3:**
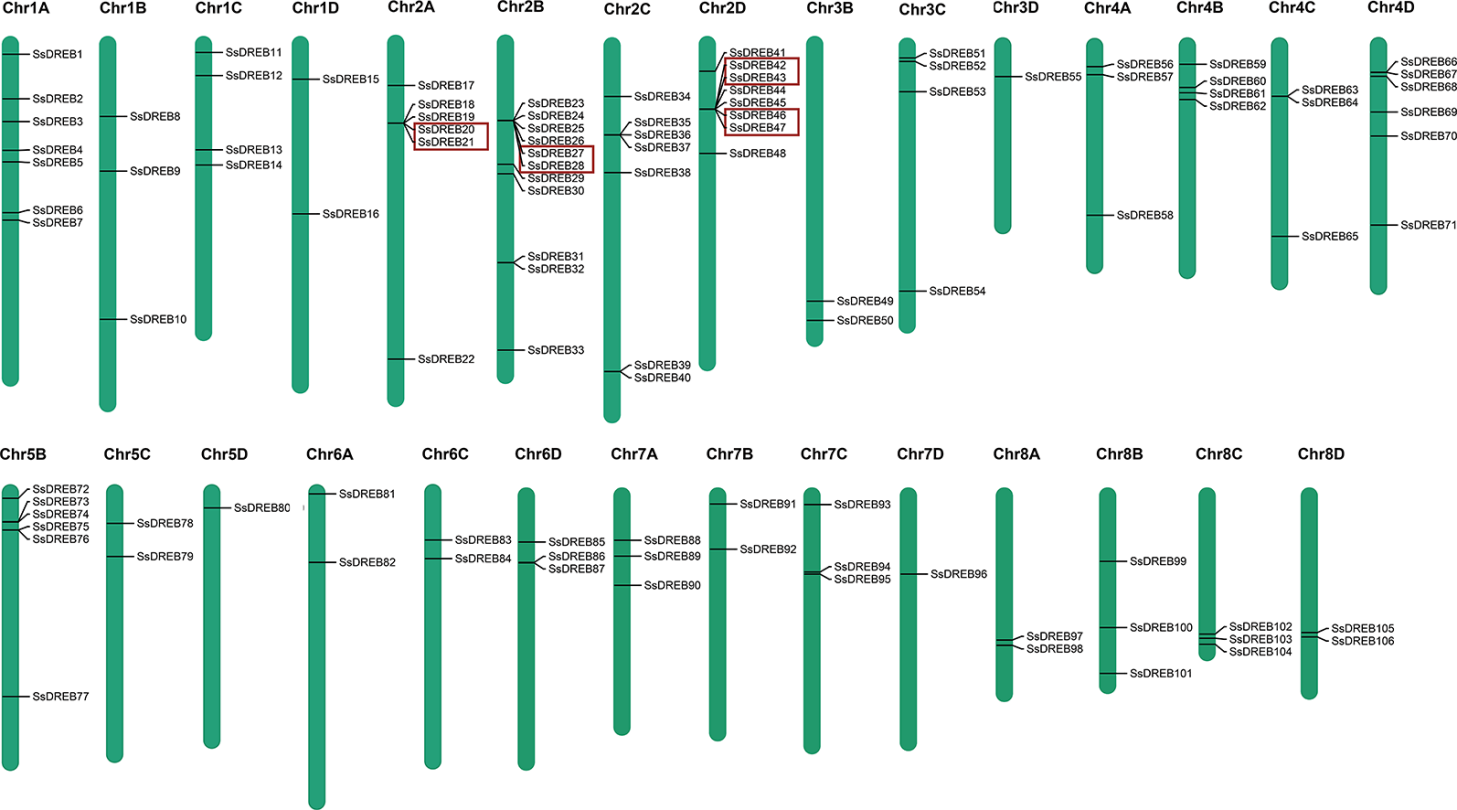
Physical locations of *SsDREB* genes in *S. spontaneum*. A total of 106 SsDREB genes were mapped onto the 29 chromosomes of *S. spontaneum*; the other four genes were identified on unassembled genomic scaffolds. The *magenta bars* represent chromosomes in *S. spontaneum*, and the scale shows the chromosome length. The gene pairs in the *red box* represent tandem duplications.

Chromosome mapping of SsDREB genes was performed using the S. spontaneum genome. Among them, 106 of the SsDREB genes are distributed across 29 chromosomes (**Figure 3**), and four SsDREBs were anchored onto four scaffolds (**Supplementary Figure S3**). Chromosome 2B contains the largest number of SsDREBs (11 SsDREB genes), whereas only one SsDREB gene is located on chromosomes 3D, 5D, and 7D. Chromosomes 3A, 5A, and 6B carry no DREB subfamily members.

**Figure 4:**
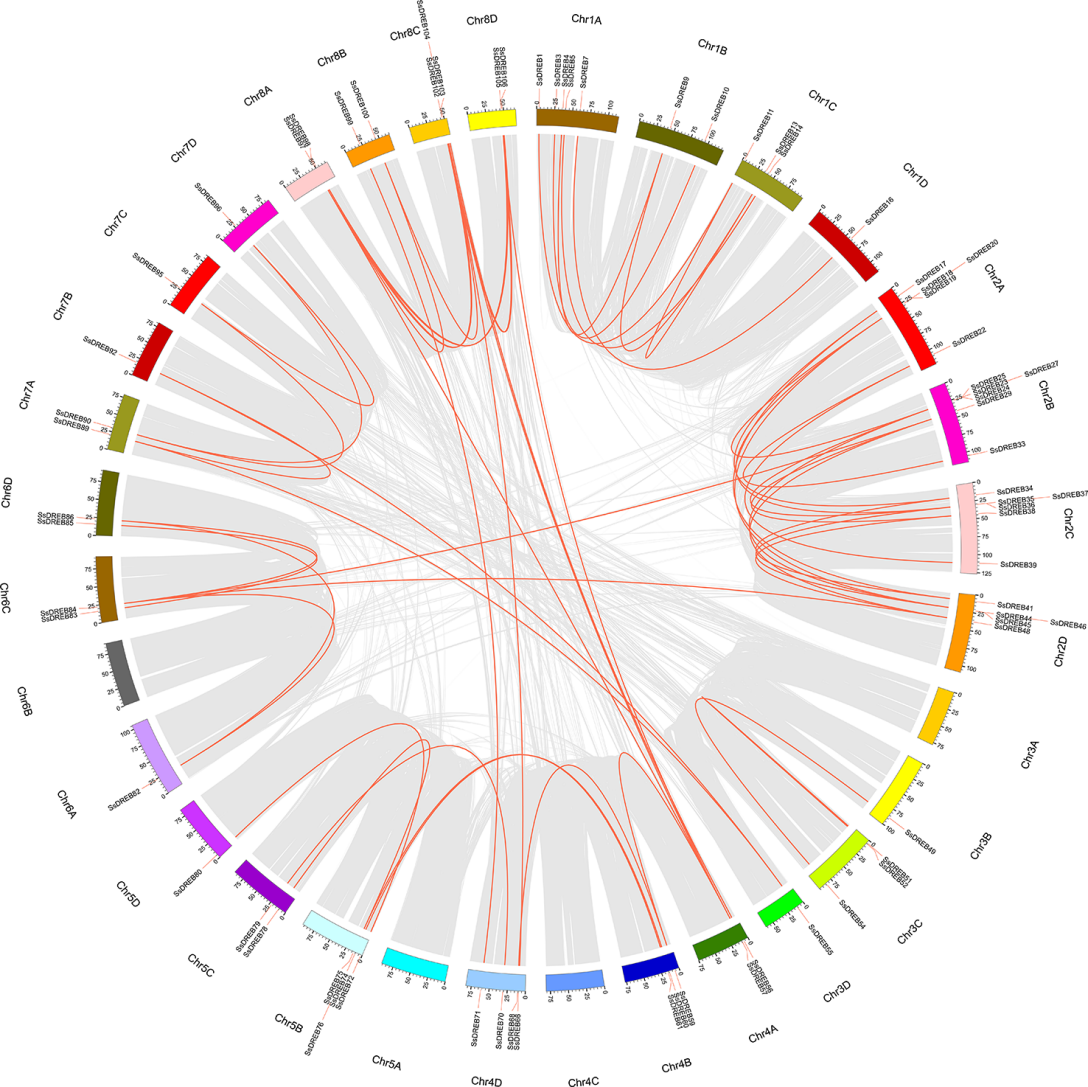
Duplications of the *SsDREB* gene subfamily. *SsDREB* genes anchored to corresponding positions on *S. spontaneum* chromosomes, as shown in *red*, were analyzed for duplications. A total of 62 pairs of duplicated 73 *SsDREB* genes are connected with gray lines.

SsDREB gene duplication events in the S. spontaneum genome were analyzed, and the results indicated segmental duplication events for 62 pairs of 73 paralogous SsDREB genes located on all chromosomes except 4C (**Figure 4**, **Table 2**, and **Supplementary Table 3**). Of these, four genes exhibited corresponding segmental replication relationships, including SsDREB57, SsDREB84, SsDREB97, and SsDREB102, located on chromosomes 4A, 6C, 8A, and 8C, respectively. There were 8, 23, and 38 SsDREB genes with three, two, and one corresponding segmental replication relationships, respectively. Furthermore, eight SsDREB genes are clustered into four tandem repeat event regions on chromosomes 2A, 2B, 2C, and 2D (**Figures 3** and **4**, **Supplementary Figure S4**, and **Supplementary Table 4**). To investigate SsDREB gene selection pressure, the non-synonymous divergence level (Ka), synonymous divergence level (Ks), and Ka/Ks ratios were calculated for these four tandem duplicated homologous gene pairs. The results suggest that three of them evolved due to negative selection, with Ka/Ks <1. In contrast, the Ka/Ks value (49.79) was far greater than 1 for the SsDREB46 and SsDREB47 pairs, highlighting that they have undergone strong positive selection.

**Table2:** Statistics of segmental duplication of *SsDREB* genes.

Number of pairs	Gene number	Gene ID
4	4	SsDREB97, SsDREB84, SsDREB57, SsDREB102
3	8	SsDREB48, SsDREB45, SsDREB35, SsDREB29, SsDREB24, SsDREB19, SsDREB105, SsDREB103
2	23	SsDREB98, SsDREB96, SsDREB95, SsDREB92, SsDREB90, SsDREB9, SsDREB89, SsDREB86, SsDREB82, SsDREB59, SsDREB55, SsDREB46, SsDREB41, SsDREB38, SsDREB37, SsDREB34, SsDREB27, SsDREB22, SsDREB17, SsDREB13, SsDREB11, SsDREB106, SsDREB100
1	38	SsDREB99, SsDREB85, SsDREB83, SsDREB80, SsDREB79, SsDREB78, SsDREB76, SsDREB75, SsDREB74, SsDREB72, SsDREB71, SsDREB70, SsDREB7, SsDREB68, SsDREB66, SsDREB61, SsDREB60, SsDREB56, SsDREB54, SsDREB52, SsDREB51, SsDREB5, SsDREB49, SsDREB44, SsDREB4, SsDREB39, SsDREB36, SsDREB33, SsDREB3, SsDREB25, SsDREB23, SsDREB20, SsDREB18, SsDREB16, SsDREB14, SsDREB104, SsDREB10, SsDREB1

**Figure 5:**
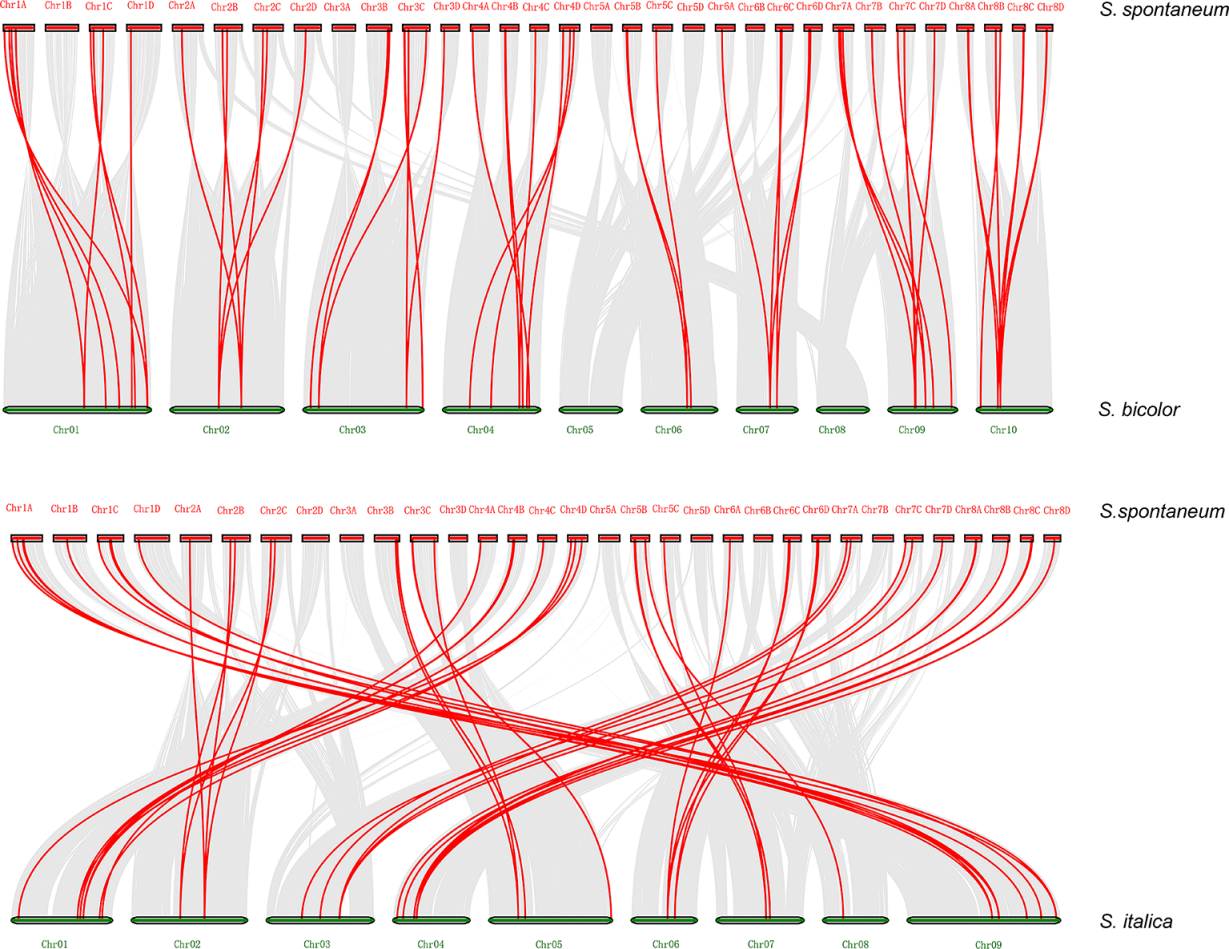
Synteny analysis between *S. spontaneum* and *S. bicolor* or *S. italica*. The *S. spontaneum* and *S. bicolor* (*S. italica*) chromosomes are represented by *red* and *green bars*, respectively, and the length of the bars roughly represents the chromosome length. The *red line* identifies direct homology between 57 *SsDREB* genes and 34 *S. bicolor* genes. In addition, 59 *SsDREB* genes exhibit a homologous relationship with 37 *DREB* genes of *S. italica*.

Synteny analysis was conducted to explore orthologous *SsDREB* gene pairs between *S. spontaneum* and four other plants from the Poaceae family. The results showed that among 110 *SsDREB* genes, 57, 59, 51, and 63 have 34, 37, 33, and 43 corresponding orthologs in *S. bicolor*, *S. italica*, *O. sativa*, and *Z. mays*, respectively (**Figure 5**, **Supplementary Figure S5**, and **Supplementary Table 5**). Among them, the highest synteny relationship was observed between *S. spontaneum* and *S. bicolor*. This finding indicates the high homology of these *SsDREB* genes and their relationships to orthologs and paralogs.

### Analysis of *cis*-Acting Elements in *SsDREB* Gene Promoters

The 2.0-kb sequence upstream from the transcriptional start site of 108 *SsDREB* genes was submitted to PlantCARE to identify *cis*-elements. All of the *cis*-elements detected in the promoter regions could be divided into three categories (**Supplementary Figure S6** and **Supplementary Table 6**). Three groups of putative *cis*-elements are related to development, hormone signaling, and environmental response, indicating that SsDREB proteins might function in these processes. Among them, 42 genes contain one or more DRE core *cis*-elements that function in responses to drought or other osmotic stresses. A total of 56 genes have one or more low-temperature response (LTR) *cis*-elements involved in low-temperature responsiveness.

Moreover, many other development- and hormone signaling-related *cis*-elements were found in the promoters of *SsDREB* genes, including the RY element (seed-specific regulation), CAT-Box (related to meristem expression), G-Box (light responsiveness), ABRE (ABA-responsive element), TGACG motif (MeJA responsiveness), and GARE motif (gibberellin-responsive element). This *cis*-element analysis illustrated that *SsDREB* genes might participate in plant development, hormone signaling, and response to environmental stress.

### Expression Profiles of *SsDREB* Genes Under Cold and Drought Stresses

**Figure 6:**
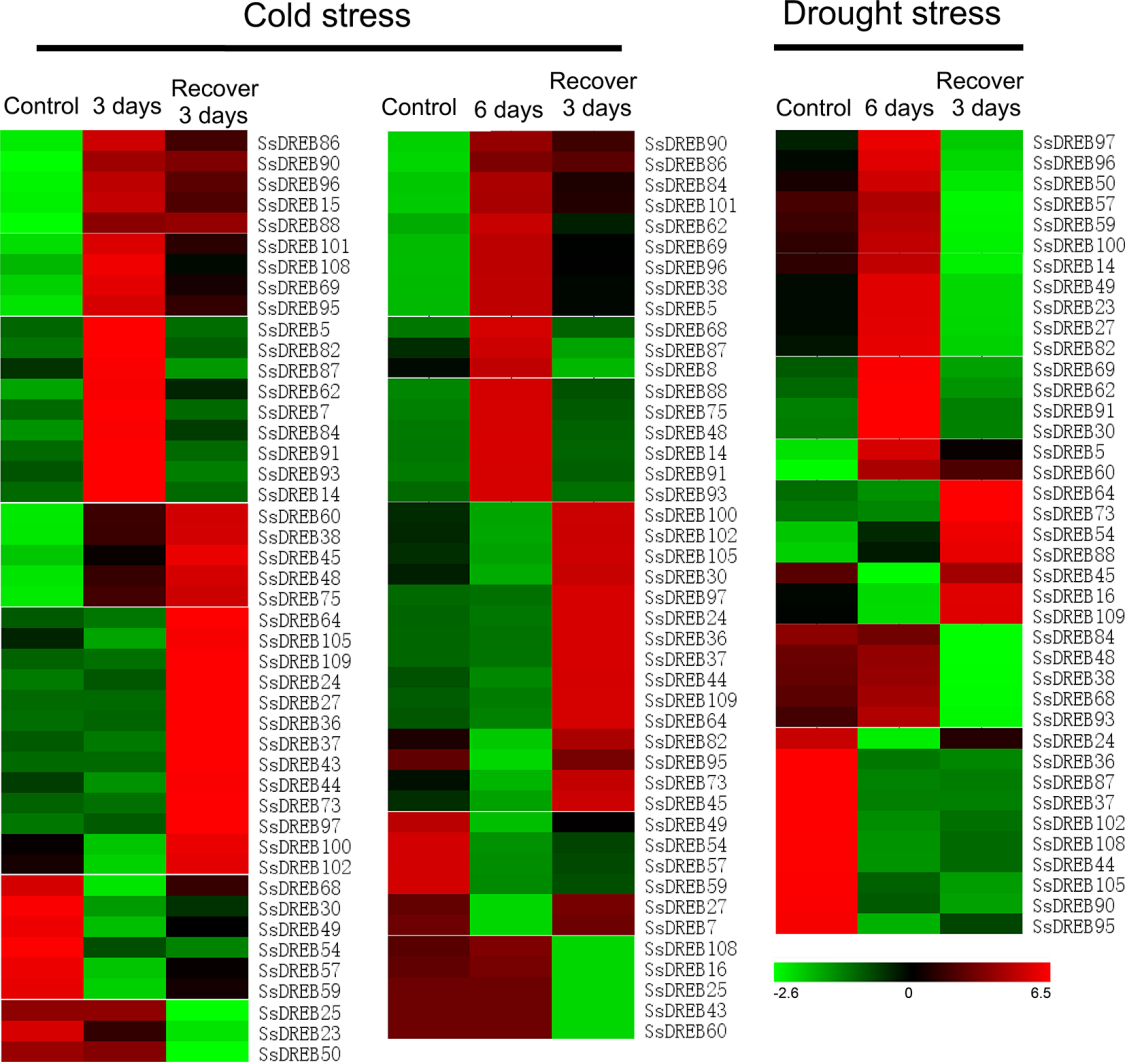
Heat map of *SsDREB* genes responding to cold and drought stresses at the transcription level. Based on the analysis of RNA-seq data, 45 genes were detected to be induced or have decreased expression under cold stress; 35 of them were up- or downregulated by drought stress. To be considered differentially expressed, the transcript must have FPKM ≥ 0.3 in at least one sample and twofold or greater change between samples (*P* ≤ 0.05), and their relative expression was normalized by log2 transformation. *Red* indicates high expression, *black* indicates intermediate expression, and *green* indicates low expression.

To investigate the putative physiological roles of SsDREB proteins, RNA-seq was performed to detect cold- and drought-induced expression of each *SsDREB* gene. As shown in **Figure 6** and **Supplementary Figure S7**, the transcriptional abundance of *SsDREB* genes varied greatly under different treatments, suggesting that *SsDREBs* have multiple functions in responses to cold and drought stress in *S. spontaneum*.

Under cold stress for 3 days, 23 *SsDREB* genes were upregulated and classified into four clusters based on the expression pattern after growth recovery for 3 days. Expression of six *SsDREB* genes was inhibited by cold stress. Three *SsDREB* genes were not induced by low temperature, but exhibited reduced expression during the recovery period. In contrast, 13 *SsDREB* genes were upregulated during growth recovery, though they were not induced by cold stress.

A total of 44 *SsDREB* genes responded to the stress of low-temperature treatment for 6 days. Expression of 18 *SsDREB* genes was upregulated, with three expression patterns in the recovery period; that is, induced expression was continuously upregulated (three *SsDREB* genes), returned to the normal growth condition level (six *SsDREB* genes), or was slightly higher than that of the control condition (nine *SsDREB* genes). In addition, expression of nine *SsDREB* genes was inhibited by cold stress treatment for 6 days, whereas expression of five *SsDREB* and 12 *SsDREB* genes did not change during 6 days of cold stress, but was suppressed and induced, respectively, during the recovery period.

There were 19 *SsDREB* genes that were upregulated by drought stress, 17 of which showed decreases to different degrees during recovery; the other two *SsDREB* genes continued to be expressed during the recovery period. In addition, 16 *SsDREB* genes were suppressed by drought treatment, with no return to the normal expression level even during the recovery period.

### Analysis of DEGs in Response to Cold and Drought Stresses

**Figure 7:**
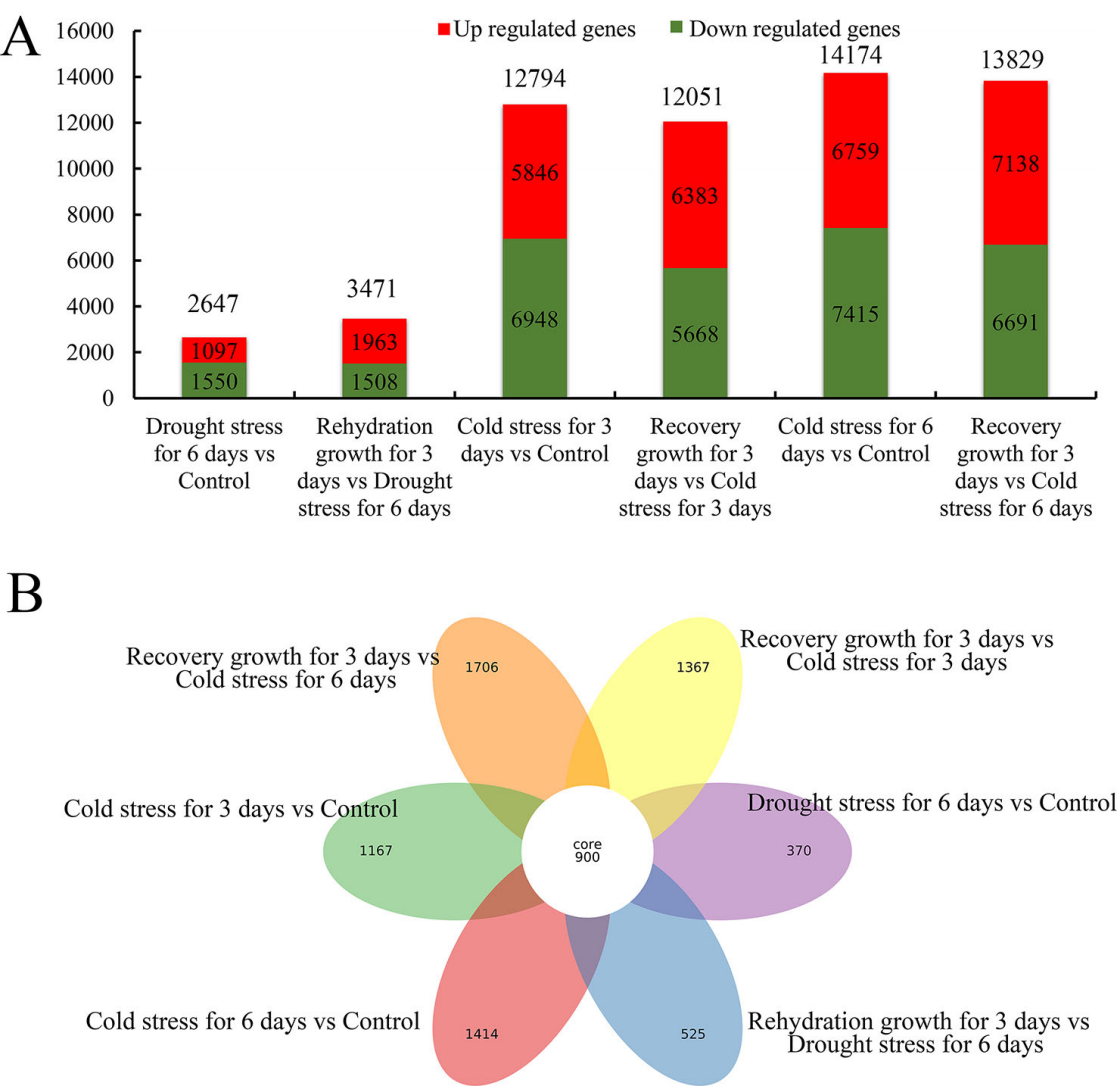
Distribution of differentially expressed genes in *S. spontaneum* responding to cold and drought stresses. **(A)** Number of downregulated, upregulated, and total differentially expressed genes (DEGs) in each comparison of *S. spontaneum* responding to cold and drought stresses. **(B)** Number of unique and common DEGs in each comparison of *S. spontaneum* responding to cold and drought stresses.

All the expressed genes with a fold change ≥2 and a false discovery rate of a corrected *P* < 0.05 were screened (**Supplementary Figure S8** and **Table 8**). As shown in **Figure 7** and **Supplementary Table 8**, 2,647 and 3,471 genes showed differential expression in *S. spontaneum* after drought stress for 4 days and rehydration for 3 days, respectively. We detected 12,749, 12,051, 14,174, and 13,829 genes with differential expression after cold stress and recovery growth after cold stress, respectively. In addition, 900 common DEGs were shared among drought and cold stress treatments. According to these results, we infer that *S. spontaneum* is more sensitive to cold than to drought stress, which is consistent with the characteristics of a tropical crop.

### Functional Categorization of Stress-Regulated Genes

GO analysis, including biological processes, cellular components, and molecular function, was used to assign functional information to the DEGs (**Supplementary Figures S9** and **S10**). For cellular components, the most enriched GO term for both the cold and drought stresses was photosystems. For biological process, the most highly represented categories were photosynthesis and related cellular processes. Regarding molecular function, the category of photosynthesis process binding was the most represented GO term, including pigment binding, chlorophyll binding, and pyridoxal phosphate binding activity. These results show that photosynthesis was the main process most seriously affected in *S. spontaneum* exposed to cold and drought stresses.

Regarding KEGG pathway enrichment annotation analysis, a large number of the DEGs mapped to categories such as carbon metabolism, carbon fixation in photosynthesis, biosynthesis of amino acids, and others, indicating that cold stress mainly affects photosynthesis mechanism and amino acid metabolism in *S. spontaneum* (**Supplementary Figures S11** and **S12**). In addition, starch and sucrose metabolism, plant hormone signal transduction, and phenylpropanoid biosynthesis, among others, representing the most enriched KEGG pathways, suggested that drought stress causes carbon metabolism and hormone responses.

### Co-Expression Networks and Co-Regulated Abiotic Stress-Responsive Putative Target Genes of SsDREBs

**Figure 8:**
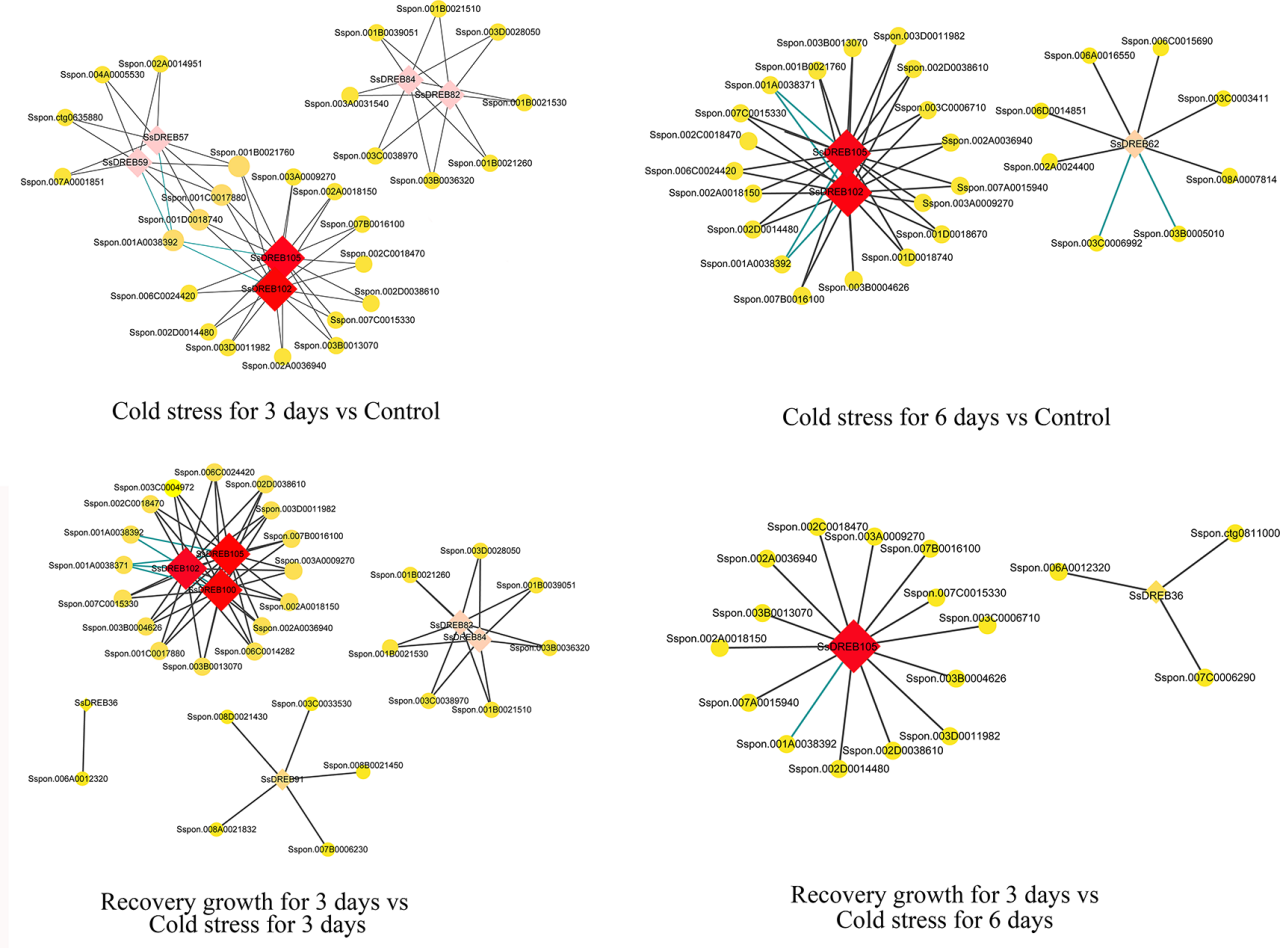
Visualization of co-expression relationships between SsDREB proteins and other DEGs in *S. spontaneum* responding to cold stress.

The identification of directly co-regulated genes is important for exploring potential transcriptional regulatory networks and putative target genes. There were four main networks containing 60 genes that function in the DREB-regulated networks of *S. spontaneum* in response to cold stress (**Figure 8** and **Supplementary Table 9**). All core SsDREB proteins, except SsDREB36 (A-1 group), belong to the A-6 group, including SsDREB100, SsDREB102, SsDREB105, SsDREB62, SsDREB57, SsDREB59, SsDREB82, and SsDREB84.

## Discussion

With the completion of sugarcane genome sequencing (Zhang et al., 2018), the function and expression of some abiotic stress factors and the entire genome of a gene family can be studied at the molecular level. We screened 110 *SsDREB* genes (**Supplementary Table 1** and **Figure 2**) using the *S. spontaneum* genome sequence.

### Comparisons of DREB Family Composition Between *S. spontaneum* and Other Plants

Evolutionary alterations have helped plants adapt to abiotic and biotic stresses by controlling a network of certain genes through modulation of specific transcription factors (Nan and Gao, 2019). Extensive studies of DREB in model plants, crops (Toshitsugu et al., 2006), or other economically important plants (Zhao et al., 2014; Xie et al., 2014; Shu et al., 2016) suggest that the number, gene structure, sequences, and functions of this subfamily differ markedly among species.

The total number of DREB superfamily genes in *S. spontaneum* is much greater than that in *Arabidopsis* (55), rice (56), *S. bicolor* (51), *Brachypodium distachyon* (54), *Z. mays* (67), grape (23), *Utricularia gibba* (42), and *A. trichopoda* (23) (**Table 1**). The genome size of these plants also differs, with 490 Mb for *Vitis vinifera*, 82 Mb for *U. gibba*, and 260 Mb for *B. distachyon*, showing that the genome size has a certain effect on the number of DREB subfamily members.

The total numbers of genes for each group were 23, 9, 5, 22, 16, and 35, corresponding to the A-1 to A-6 groups (**Table 1**). The number of *DREB* genes belonging to A-3 in *A. trichopoda, Arabidopsis, Fragaria vesca*, and *Z. mays* is only one, and it is zero in *B. distachyon* and *V. vinifera*. In addition, the gene numbers in this group in apple (2) (Shen et al., 2017), *Populus trichocarpa* (2) (Chen et al., 2013), and *Salix arbutifolia* (2) (Rao et al., 2015) are fewer than in *S. italica* (18) (Shi et al., 2018a). Regarding *S. spontaneum*, the number of genes in A-3 is 2.5 times that of *S. bicolor*. Similar duplications have also occurred in other groups, especially for the A-1 and A-6 groups, which are almost four times as large as in sorghum. Thus, we propose that the expansion of groups A-1, A-3, and A-6 is also an important factor leading to the increase in the SsDREB family.

Duplications of chromosomal fragments or entire genomes have been considered to be the major sources of evolution, including producing new gene functions and expression patterns (Guo et al., 2016; Zhang and Li, 2018); segmental duplications produce homologous genes, which expand the total gene number (Shu et al., 2016). In total, we identified 62 paralogous pairs (including 73 *SsDREB* genes; **Supplementary Table 3**) confirmed to be mainly produced by segmental duplication in *S. spontaneum* (**Figure 4**). In contrast, only four homologous pairs were produced through tandem repeats. Moreover, gene pairs with tandem duplications are all located on chromosomes of the same origin, namely, chromosome 2.

Paralogous numbers of *AP2/ERF* genes in several plant species have been reported in rice (41), grape (76), *Arabidopsis* (51), and Chinese jujube (18), which contain AP2, RAV, ERF, and DREB subfamilies. All were much lower than in *S. spontaneum*. Except for group A-3, gene pairs in all groups were found; 24 gene pairs belonging to group A-6 accounted for 38.71%, followed by the A-1, A-4, A-5, and A-2 groups, containing 13, 12, 8, and 5 gene pairs of segmental duplication, respectively (**Supplementary Table 3**).

Previous reports have revealed that some transcription factors with similar conserved sequences are located on the same chromosome in Tartary buckwheat (Liu et al., 2019), and similar patterns have also been found in *A. thaliana* (Sakuma et al., 2002) and *V. vinifera* (Licausi et al., 2010), which are thought to represent homologous fragments caused by ancestral polyploidy events. However, there were no such characteristics in *S. spontaneum*. Combining the results of interspecies synteny with sorghum, we inferred that expansion of the SsDREB subfamily was mainly due to segmental duplication along with the WGD of *S. spontaneum*. These results suggest that the DREB subfamily in *S. spontaneum* has undergone an unevenly significant expansion in each group, which was driven by the WGD.

*DREB* genes were initially thought to be intronless. In recent years, many *DREB* genes with introns have been found, especially in Poaceae family plants, such as barley (Guo et al., 2016), rice (Matsukura et al., 2010), maize (Qin et al., 2007; Liu et al., 2013), wheat (Sazegari and Niazi, 2012), *Leymus chinesis* (Liu et al., 2017b), and others, as well as in legumes. However, *DREB* genes in *Arabidopsis* and other Cruciferae plants have no introns. Additionally, the existence of introns leads to alternative splicing, especially in the A-2 group (Qin et al., 2007; Matsukura et al., 2010; Liu et al., 2017b). In our study, structural analysis of the DREB subfamily showed that 70.9% of *SsDREB* genes (78) have no introns, though the number of introns in the other 32 genes ranges from one to six. As diversity of *SsDREB* gene structures will make gene expression and regulation more complex and flexible, the evolutionary process and mechanism of producing *DREB* gene structures requires further systematic analysis.

### The Co-Expression and Interaction Network of SsDREBs Under Cold and Drought Stresses

We identified 23 and 18 genes that were upregulated during cold stress for 3 and 6 days, respectively (**Figure 6** and **Supplementary Figure S7**), and most of them returned to control or close to control levels after resuming culture for 3 days. These results suggested that these *SsDREB* genes play positive roles during cold stress in *S. spontaneum*. Among them, *SsDREB75*, *SsDREB84*, and *SsDREB101* showed obviously induced expression. Furthermore, such induced expression is supported by the results of *cis*-element analysis, namely, the presence of LTR elements in promoter regions (**Supplementary Table 7** and **Supplementary Figure S6**). In contrast, nine genes were downregulated by cold stress after 3 days, and nine genes were simultaneously downregulated after 6 days of cold stress, which were considered to have a negative function during the response to cold stress. Moreover, some *SsDREB* genes showed opposite expression patterns between the 3- and 6-day cold treatments, such as *SsDREB95*.

Regarding drought stress, only seven genes were obviously upregulated compared with 21 genes showing reduced expression. Interestingly, *SsDREB5*, *SsDREB88*, and *SsDREB90* were upregulated by both cold and drought stresses. These *SsDREB* genes expressed in response to both cold and drought stresses belong to the A-1 to A-6 groups. The promoter of some *SsDREB* genes that respond to stress contain cold or drought stress response elements, whereas others do not contain such elements, reflecting the complexity of the mechanism of regulation of *SsDREB* genes involved in cold and drought stress tolerance. This might be related to WGD, evolution, and adaptation to the environment in *S. spontaneum*.

The cold and drought stress response and regulated DREB pathways have been extensively explored. MKK4/5-MPK3/6 is activated by cold stress, phosphorylates ICE1, promotes its degradation, decreases the transcription level of CBFs, and negatively regulates the cold stress response (Zhao et al., 2017; Shi et al., 2018b). CRPK1 is modified by low-temperature-induced phosphorylation of 14-3-3, and phosphorylated 14-3-3 is transported from the cytoplasm to the nucleus and involved in ubiquitination and degradation of CBF/DREB (Liu et al., 2017a). In *Arabidopsis*, DRIP1 interacts with DREB2A (a member of the A-2 group) and functions as a RING E3 ligase that is capable of mediating DREB2A ubiquitination to negatively regulate plant drought stress-responsive gene expression (Qin et al., 2008). CBF1 can modulate the accumulation of growth-repressing DELLA proteins *via* its effect on gibberellin metabolism (Achard et al., 2008). Transgenic expression of *LcDREB2a* can improve drought and salt tolerance (Peng et al., 2011). In rice, COLD1 interacts with RGA to activate Ca^2+^ channels, which in turn stimulate the CBF transcription factor regulatory pathway (Ma et al., 2015).

In recent studies, RNA-seq has revealed that signal regulation by transcription factors, metabolic pathways including synthesis of carbohydrate, secondary metabolites, and antioxidant enzymes play vital roles in responses to cold stress in *Casuarina equisetifolia* (Li et al., 2016a). Moreover, physiologic and transcriptomic analyses have confirmed that M54, a chilling-tolerant maize strain, achieves a relatively high cold tolerance through photosystem protection and cell membrane stability maintenance (Li et al., 2019). In total, 933, 1,644, and 133 stress tolerance genes were identified after osmotic, cold, and salt treatments in banana, respectively. Further integrated analyses showed that 30 tolerance genes, including transcription factors and E3 ubiquitin protein ligases, can be regulated by osmotic, cold, and salt stresses (Hu et al., 2017).

Co-expression and co-network methods are useful for high-throughput expression profiling data analysis, especially when the goal is to elucidate relationships among related genes. To further understand the function of SsDREB proteins in *S. spontaneum* in response to cold and drought stresses, we constructed co-expression and co-regulated networks of DEGs only related to SsDREBs. The results showed four co-networks for cold stress, but no co-networks for drought stress. With regard to their co-expression and interacting genes for 3 days of cold stress, genes encoding a MYB-like protein (4), WRKY protein (6), choline/ethanolamine kinase (3), sugar transporter (1), and glycosyltransferase (1) are mainly regulated *via* SsDREB102 and SsDREB105 or interaction with SsDREB82 and SsDREB84. Similar co-expression and co-regulated networks can be discovered by comparison of recovery growth for 3 days vs. cold stress for 3 days. When treated with 6 days of cold stress, many genes encoding an E3 ubiquitin-protein ligase (18) were identified as interacting with SsDREB102 and SsDREB105. Regarding comparison of growth recovery for 3 days vs. cold stress for 6 days, genes encoding a BTB/POZ protein, MYB-like protein, WRKY protein, choline/ethanolamine kinase, and protein tyrosine kinase were mainly co-regulated with SsDREB105.

According to our results, we propose that in *S. spontaneum*, more than six SsDREB proteins together with other transcriptional factors regulate the early response to cold stress. In addition, SsDREB102 and SsDREB105 play important and positive roles in E3-mediated ubiquitination in response to long-term cold stress in *S. spontaneum*.

## Conclusion

In this study, 110 *SsDREB* genes were identified in *S. spontaneum* and were clearly divided into six groups based on phylogenetic analysis. Our results suggest that the DREB subfamily in *S. spontaneum* has undergone an unevenly significant expansion, which was driven by WGD. In addition, sequence analysis revealed that 32 *SsDREB* genes possess two to seven exons, and many have several *cis*-elements in their promoter region that are related to development, phytohormone, and environmental stress responses. Furthermore, 45 *SsDREB* members were mainly expressed in response to cold stress, 35 of which were also up- or downregulated by drought stress, accounting for less than half. Most importantly, *SsDREB100, SsDREB102*, and *SsDREB105* are proposed as key *SsDREB* genes that play key roles in tolerance to cold stress and will be important candidate genes for future breeding improvement. Overall, our work delineates the evolutionary relationship and expression patterns of *DREB* genes in *S. spontaneum*, and this information will provide fundamental insight into the precise function of DREB for further research.

## Data Availability Statement

The datasets generated for this study have been deposited in the NCBI (PRJNA549834).

## Author Contributions

Data curation: XS, BZ, CL and YoL. Formal analysis: BZ, CL, LQ and YF. Funding acquisition: YaL and JW. Investigation: ZZ and HZ. Project administration: YaL and JW. Writing-original draft: XH and RC. Writing-review & editing: XH, XS and LP. All authors approved the final manuscript.

## Funding

This research was funded by the National Natural Science Foundation of China (31660356, 31360312, 31701363), Development and Regulation of Economically Important Traits in Tropical Crops (2018YFD1000500), Guangxi Science and Technology Base and Talents Special Project (GuikeAD17195100), Fund for Guangxi Innovation Teams of Modern Agriculture Technology (nycytxgxcxtd-03-01), the Natural Science Foundation of Guangxi Province (2015GXNSFBA139011), and Fund Science and Technology Development of Guangxi Academy of Agricultural Sciences (Guinongke2017JZ19, Guinongke2016JM06). The authors thank Biomics (Beijing) Biotech Co. Ltd. for providing some help in the RNA-seq analysis.

## Conflict of Interest

The authors declare that the research was conducted in the absence of any commercial or financial relationships that could be construed as a potential conflict of interest.

## Supplementary Material

Supplementary Figure S1 Display of conserved motifs of SsDREB proteins. The conserved motifs in DREB proteins of *S. spontaneum* were searched using Multiple Expectation Maximization for Motif Elicitation (MEME) Suite version 5.0.4. The overall height in each stack indicates the sequence conservation at that position; the height of each residue letter indicates the relative frequency of the corresponding residue.

Supplementary Figure S2 Phylogenetic analysis of the DREB family. Evolutionary analysis was conducted in MEGA X, with 1000 bootstrap replicates. A simplified version of the neighbor-joining (NJ) tree is displayed, with proteins from *S. italic*, *S. bicolor*, *Z. mays*, *O. sativa*, *A. trichopoda*, and Arabidopsis, which were downloaded from the plant transcription factor database (http://planttfdb.cbi.pku.edu.cn/). The species used in the phylogenetic tree is shown in **Table 2**.

Supplementary Figure S3 Physical locations of four *SsDREB* genes in the unassembled genomic scaffolds of *S. spontaneum*.

Supplementary Figure S4 Distribution of four pairs of tandem repeat events for eight *SsDREB* genes on chromosome.

Supplementary Figure S5 Synteny analysis between *S. spontaneum* and *Z. mays* or *O. sativa*. The *S. spontaneum* and *Z. mays* (or *O. sativa*) chromosomes are represented by red and green bars, respectively, and the length of the bars roughly represents the chromosome length. The red line identifies direct homology between 63 *SsDREB* genes and 43 *Z. mays* genes. In addition, 51 *SsDREB* genes have a homologous relationship with 33 *DREB* genes of *O. sativa*.

Supplementary Figure S6 *Cis*-regulatory element analysis of *SsDREB* genes. The promoter regions of *SsDREB* genes were analyzed using the PlantCARE database (http://bioinformatics.psb.ugent.be/webtools/plantcare/html/). The various colored bars represent different *cis*-acting elements, as shown in the legend.

Supplementary Figure S7 Cluster analysis of differentially expressed *SsDREB* under cold and drought stresses. The pink line represents the expression trend of the cluster. The gray line represents the expression profile of every gene.

Supplementary Figure S8 The volcano plot of all genes in *S. spontaneum* expressed in response to cold and drought treatments.

Supplementary Figure S9 GO annotations of DEGs in *S. spontaneum* in response to cold and drought treatments.

Supplementary Figure S10 Top 20 most enriched functional groups under GO categories for DEGs in *S. spontaneum* in response to cold and drought treatments.

Supplementary Figure S11 KEGG pathway enrichment annotation of DEGs in *S. spontaneum* in response to cold and drought treatments.

Supplementary Figure S12 The most enriched functional groups under KEGG pathway enrichment annotation for DEGs in *S. spontaneum* in response to cold and drought treatments.

Supplementary Table 1 *SsDREB* gene information obtained in this study.

Supplementary Table 2 The gene ID and sequences of the conserved AP2 domain used for constructing the phylogenic tree and comparisons of gene numbers in each group.

Supplementary Table 3 Gene pairs with segmental duplication of *SsDREB* genes.

Supplementary Table 4 Ka/Ks analysis for tandem duplicated *SsDREB* gene pairs in *S. spontaneum*.

Supplementary Table 5 The location and gene ID of *SsDREB* genes and their orthologs in *S. italica*, *S. bicolor*, *Z. mays* and *O. sativa*.

Supplementary Table 6 The detailed distribution of *cis*-regulatory elements for *SsDREB* genes.

Supplementary Table 7 FPKM values of transcriptional levels of *SsDREB* genes responding to cold and drought stresses.

Supplementary Table 8 Counts of reads, FPKM values and functional annotations of DEGs responding to cold and drought stresses.

Supplementary Table 9 List of DEGs screened *via* protein-protein interaction analysis.

## Abbreviations

DRE, dehydration-responsive element; DREB, dehydration-responsive element-binding protein; CRT, C-repeat; LTR, low-temperature responsiveness; CBF, C-repeat-binding factors; COR, cold-regulated; ERF, ethylene-responsive element binding factors.
