# Supplementary figures and images for: Genome-Wide Analysis of the DREB Subfamily in Saccharum spontaneum Reveals Their Functional Divergence During Cold and Drought Stresses
Source: Front Genet. 2020 Feb 5;10:1326. doi: 10.3389/fgene.2019.01326 (PMC7013043; doi:10.3389/fgene.2019.01326)

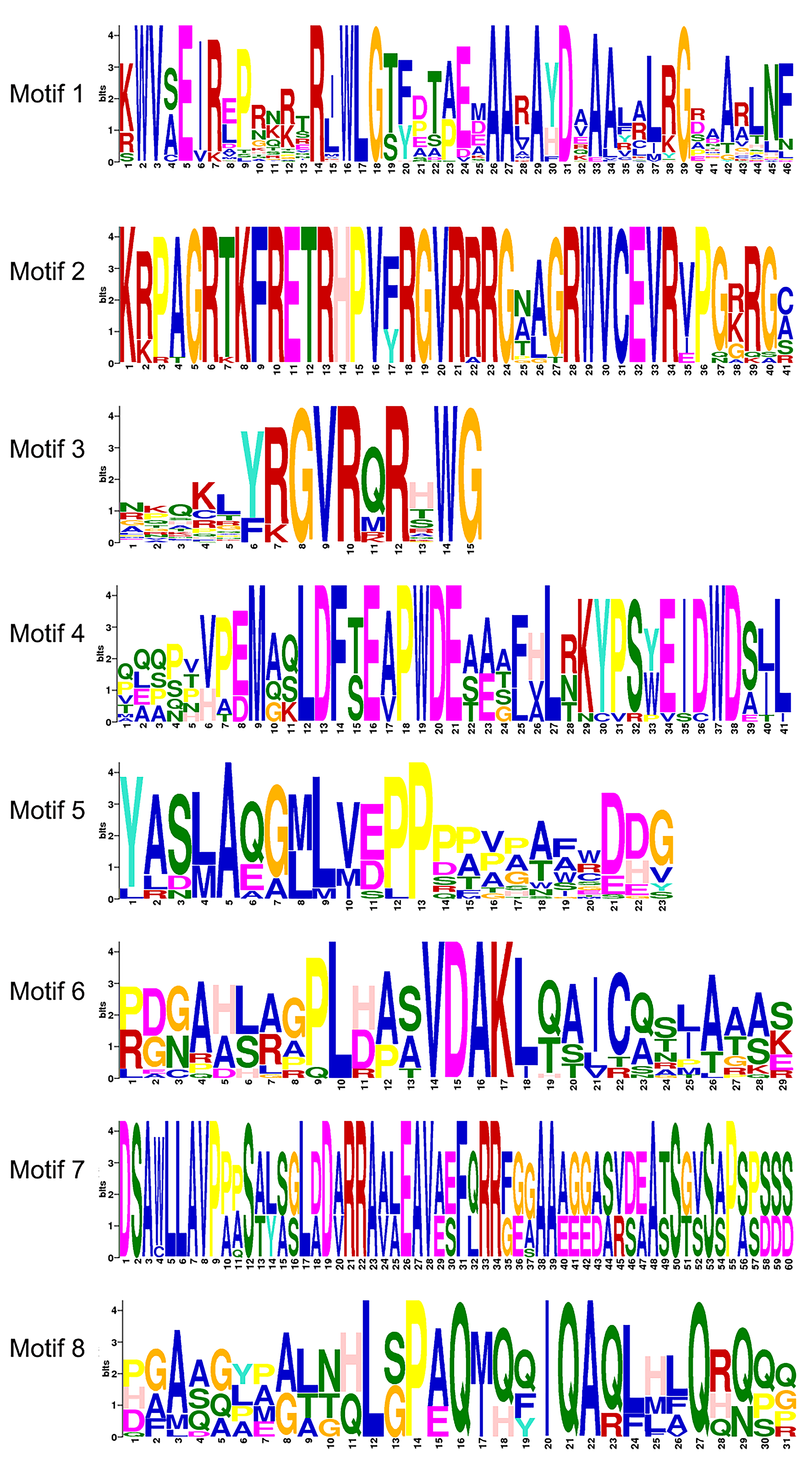

Supplement: Supplementary Figure S1 — Display of conserved motifs of SsDREB proteins. The conserved motifs in DREB proteins of S. spontaneum were searched using Multiple Expectation Maximization for Motif Elicitation (MEME) Suite version 5.0.4. The overall height in each stack indicates the sequence conservation at that position; the height of each residue letter indicates the relative frequency of the corresponding residue. [file Image_1.tif]

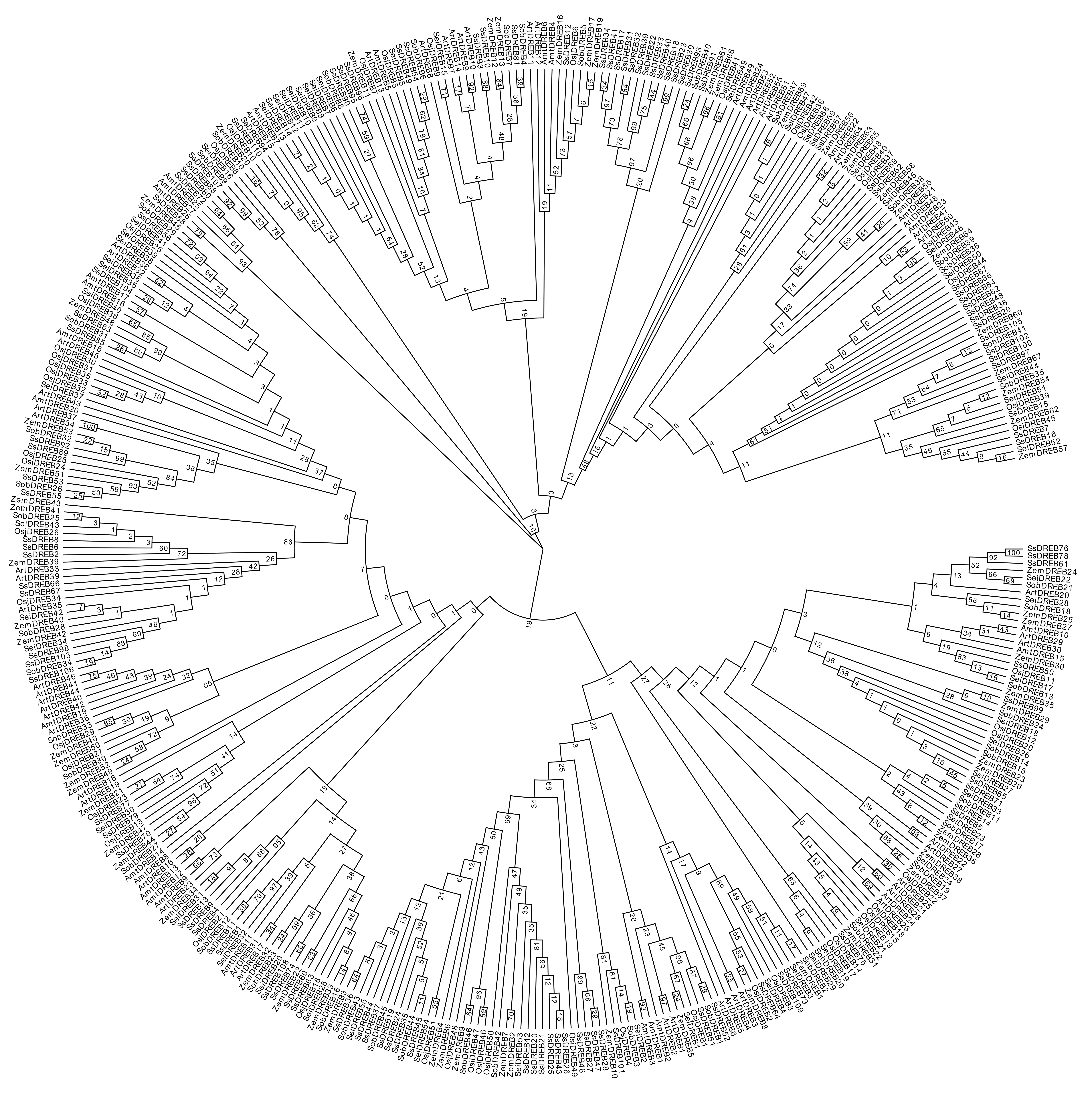

Supplement: Supplementary Figure S2 — Phylogenetic analysis of the DREB family. Evolutionary analysis was conducted in MEGA X, with 1000 bootstrap replicates. A simplified version of the neighbor-joining (NJ) tree is displayed, with proteins from S. italic, S. bicolor, Z. mays, O. sativa, A. trichopoda, and Arabidopsis, which were downloaded from the plant transcription factor database (http://planttfdb.cbi.pku.edu.cn/). The species used in the phylogenetic tree is shown in Table 2. [file Image_2.tif]

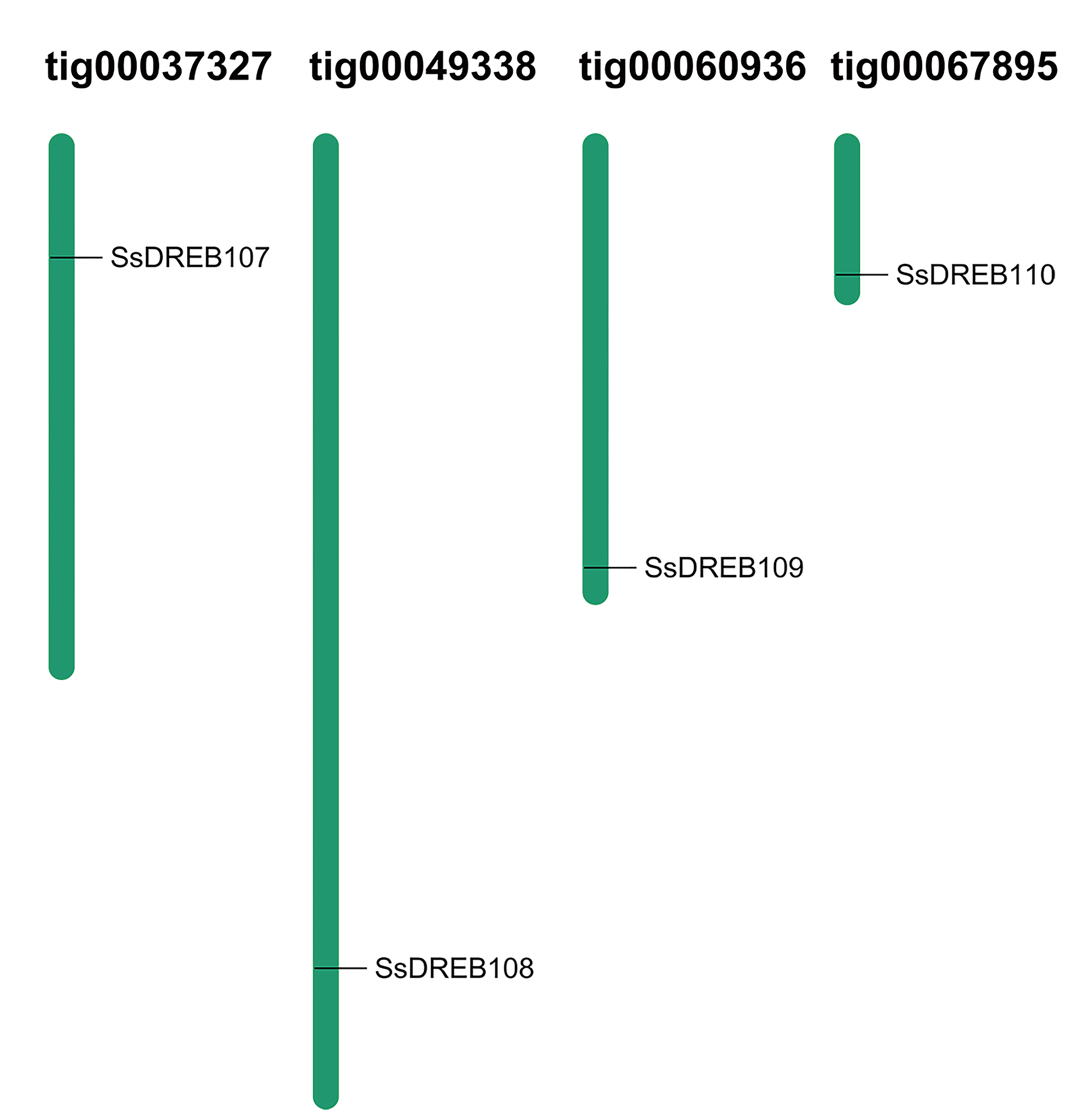

Supplement: Supplementary Figure S3 — Physical locations of four SsDREB genes in the unassembled genomic scaffolds of S. spontaneum. [file Image_3.tif]

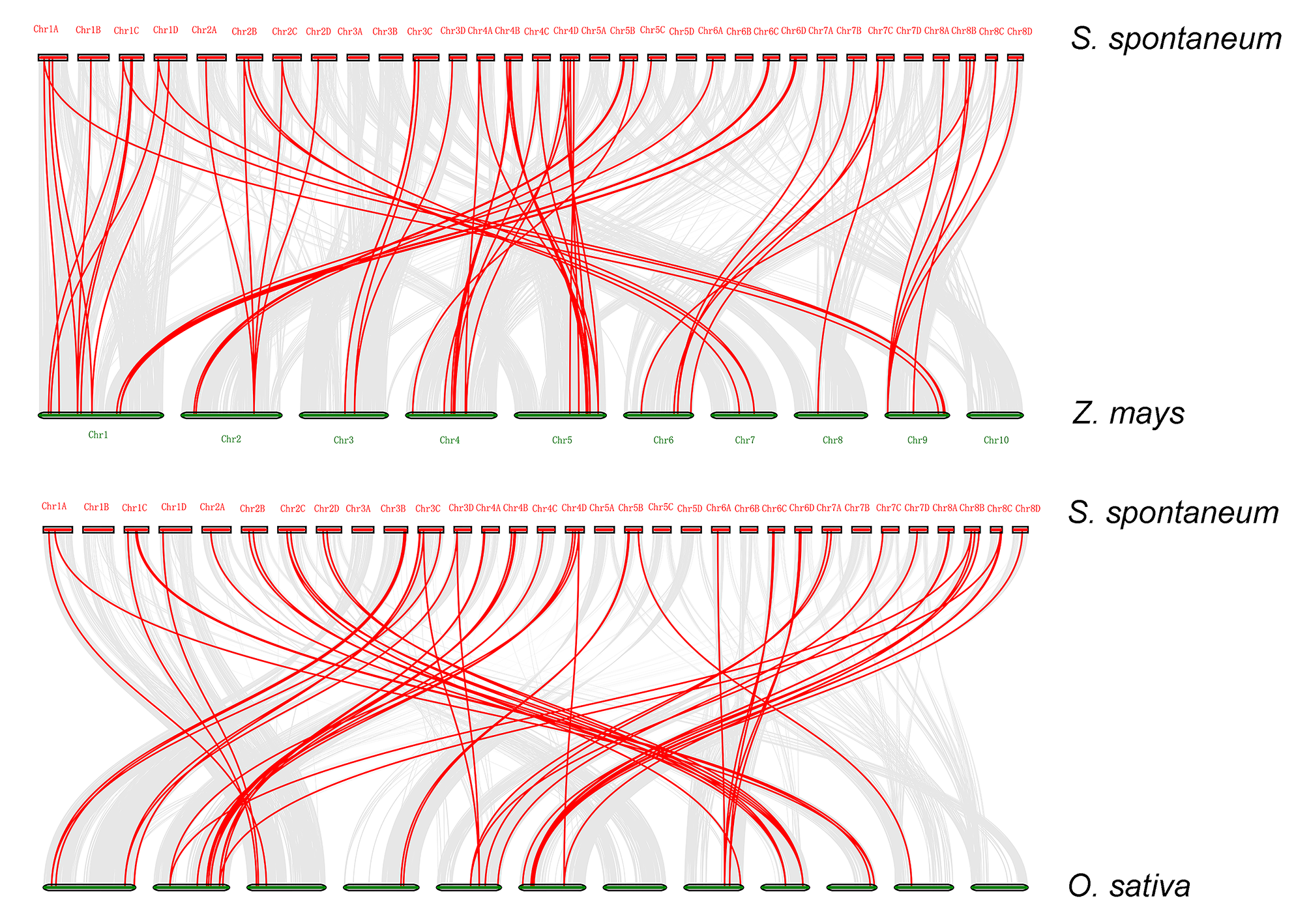

Supplement: Supplementary Figure S5 — Synteny analysis between S. spontaneum and Z. mays or O. sativa. The S. spontaneum and Z. mays (or O. sativa) chromosomes are represented by red and green bars, respectively, and the length of the bars roughly represents the chromosome length. The red line identifies direct homology between 63 SsDREB genes and 43 Z. mays genes. In addition, 51 SsDREB genes have a homologous relationship with 33 DREB genes of O. sativa. [file Image_5.tif]

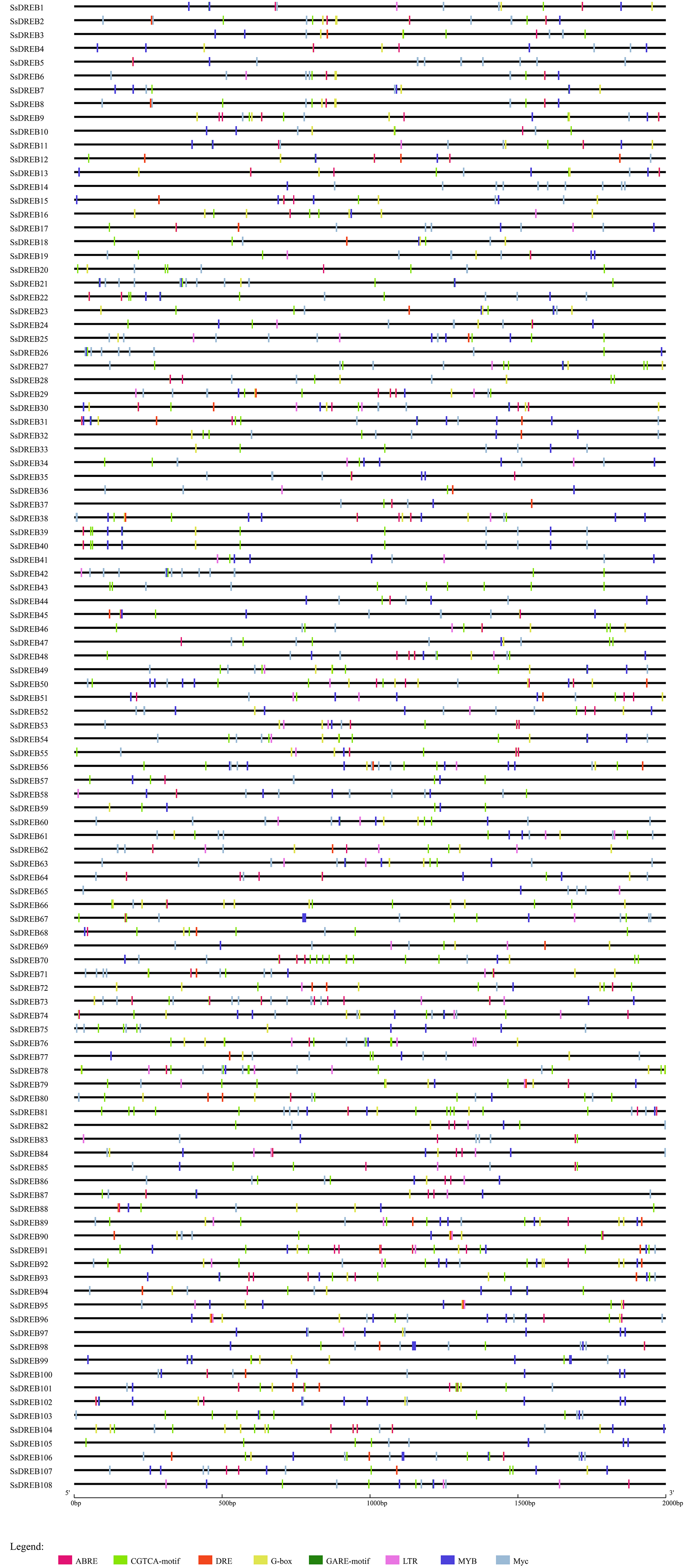

Supplement: Supplementary Figure S6 — Cis-regulatory element analysis of SsDREB genes. The promoter regions of SsDREB genes were analyzed using the PlantCARE database (http://bioinformatics.psb.ugent.be/webtools/plantcare/html/). The various colored bars represent different cis-acting elements, as shown in the legend. [file Image_6.tif]

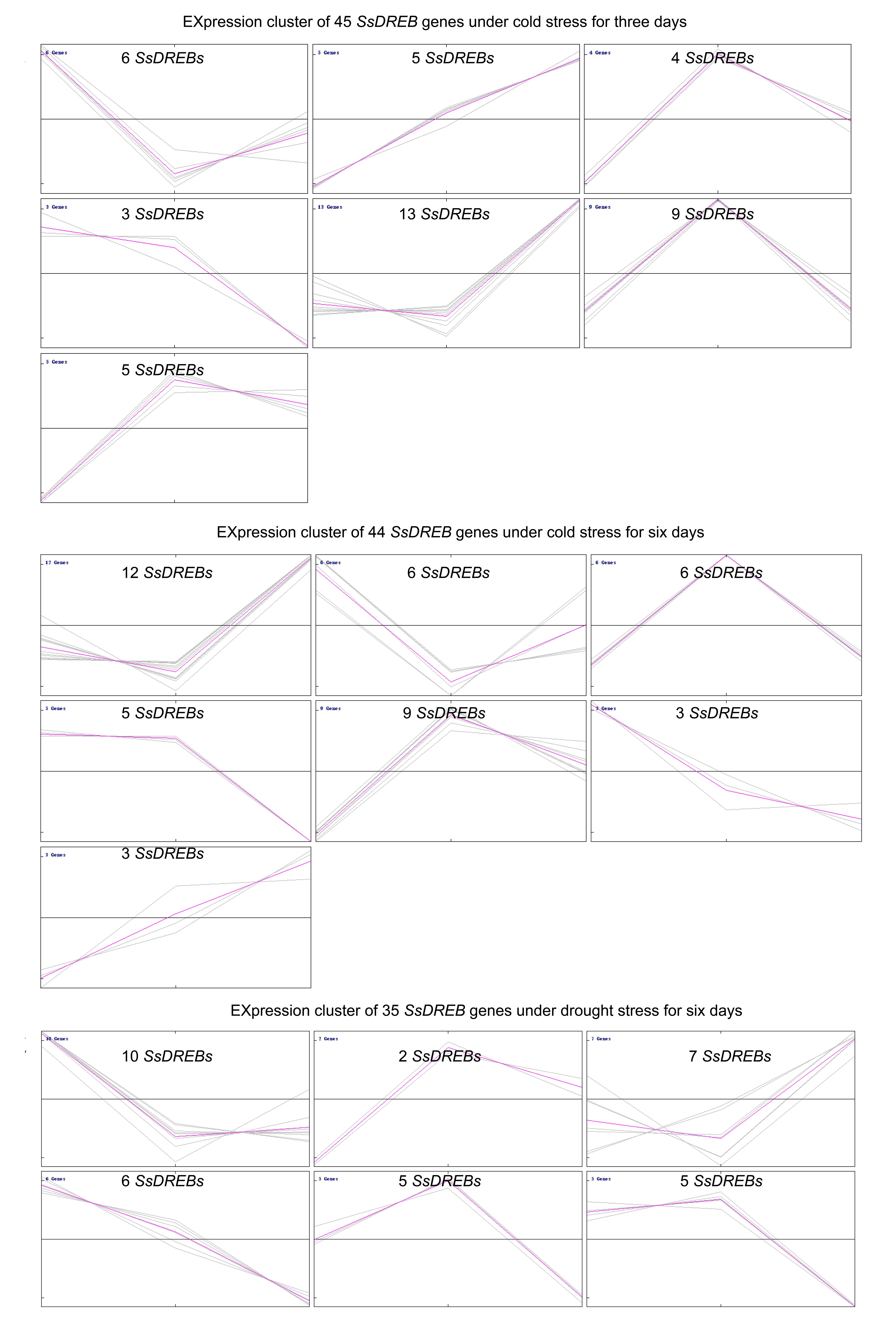

Supplement: Supplementary Figure S7 — Cluster analysis of differentially expressed SsDREB under cold and drought stresses. The pink line represents the expression trend of the cluster. The gray line represents the expression profile of every gene. [file Image_7.tif]

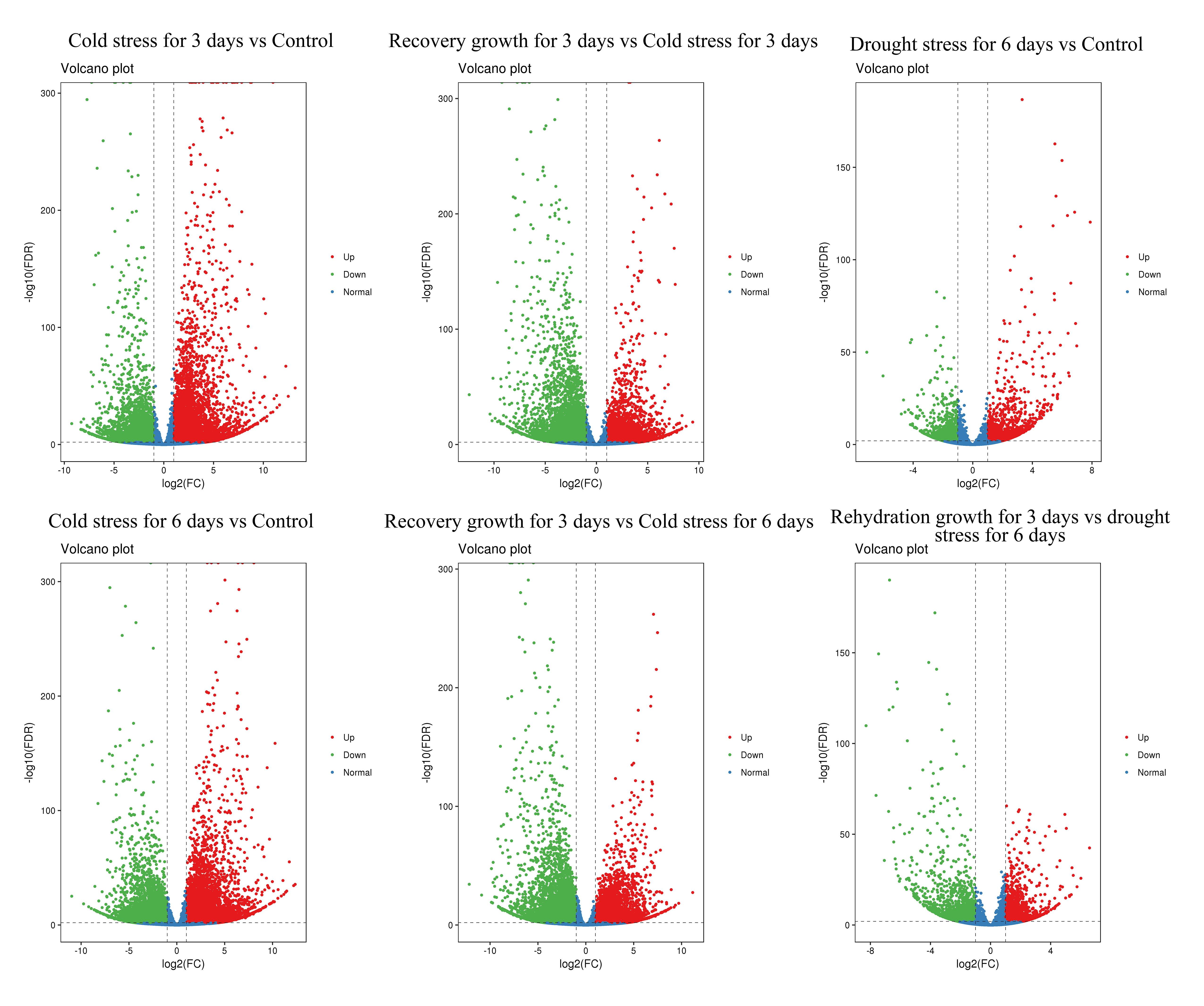

Supplement: Supplementary Figure S8 — The volcano plot of all genes in S. spontaneum expressed in response to cold and drought treatments. [file Image_8.tif]

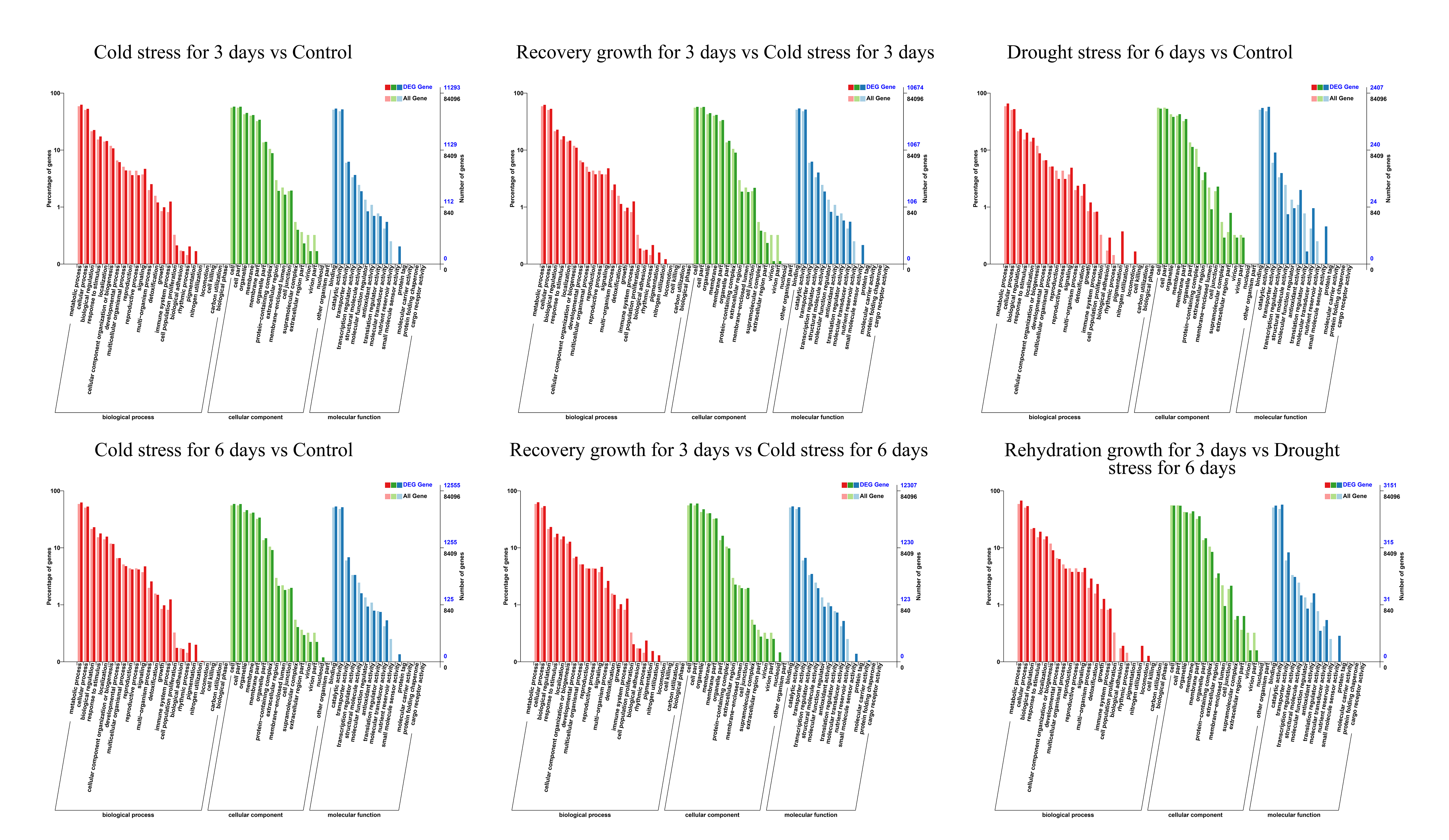

Supplement: Supplementary Figure S9 — GO annotations of DEGs in S. spontaneum in response to cold and drought treatments. [file Image_9.tif]

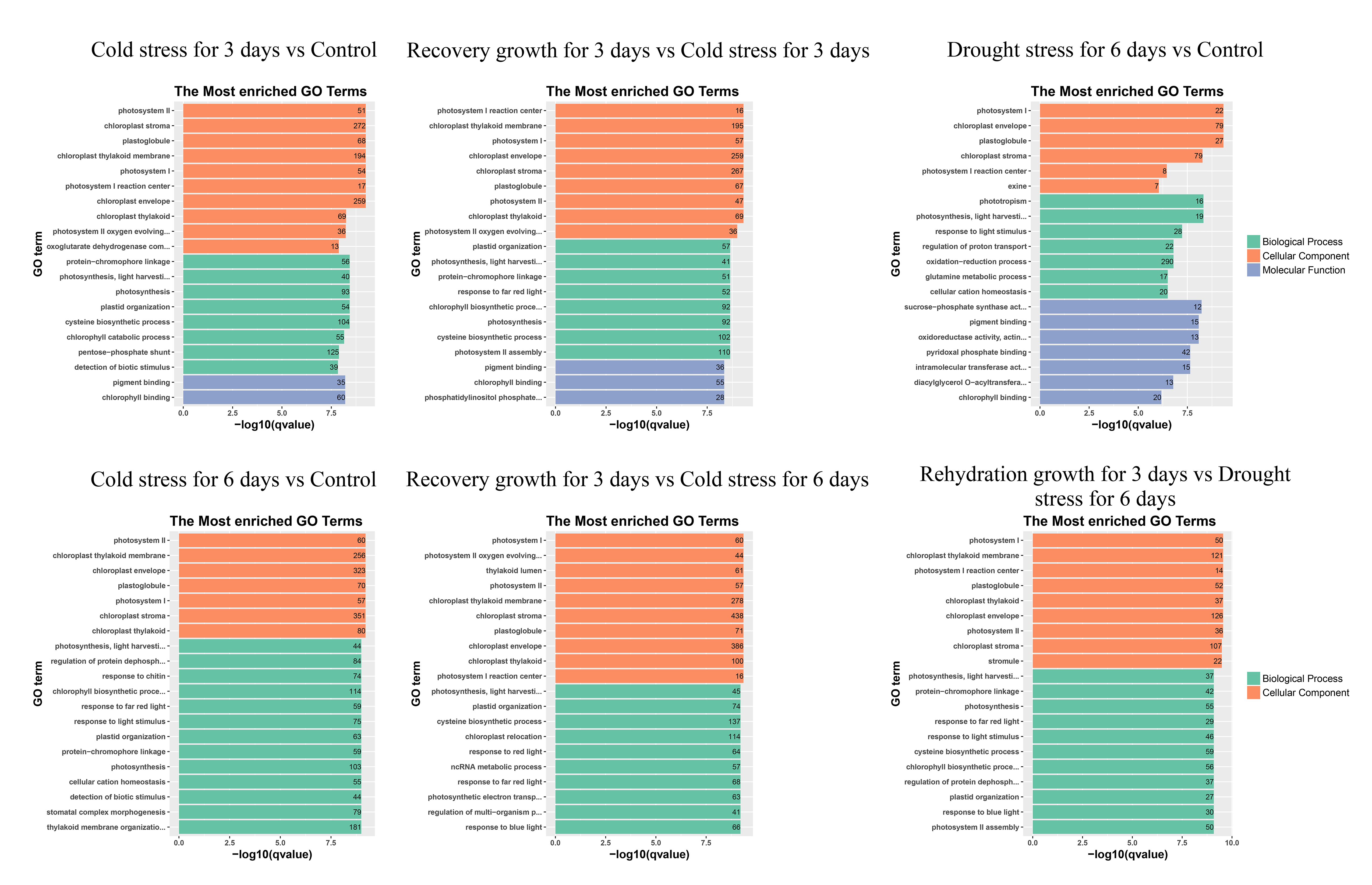

Supplement: Supplementary Figure S10 — Top 20 most enriched functional groups under GO categories for DEGs in S. spontaneum in response to cold and drought treatments. [file Image_10.tif]

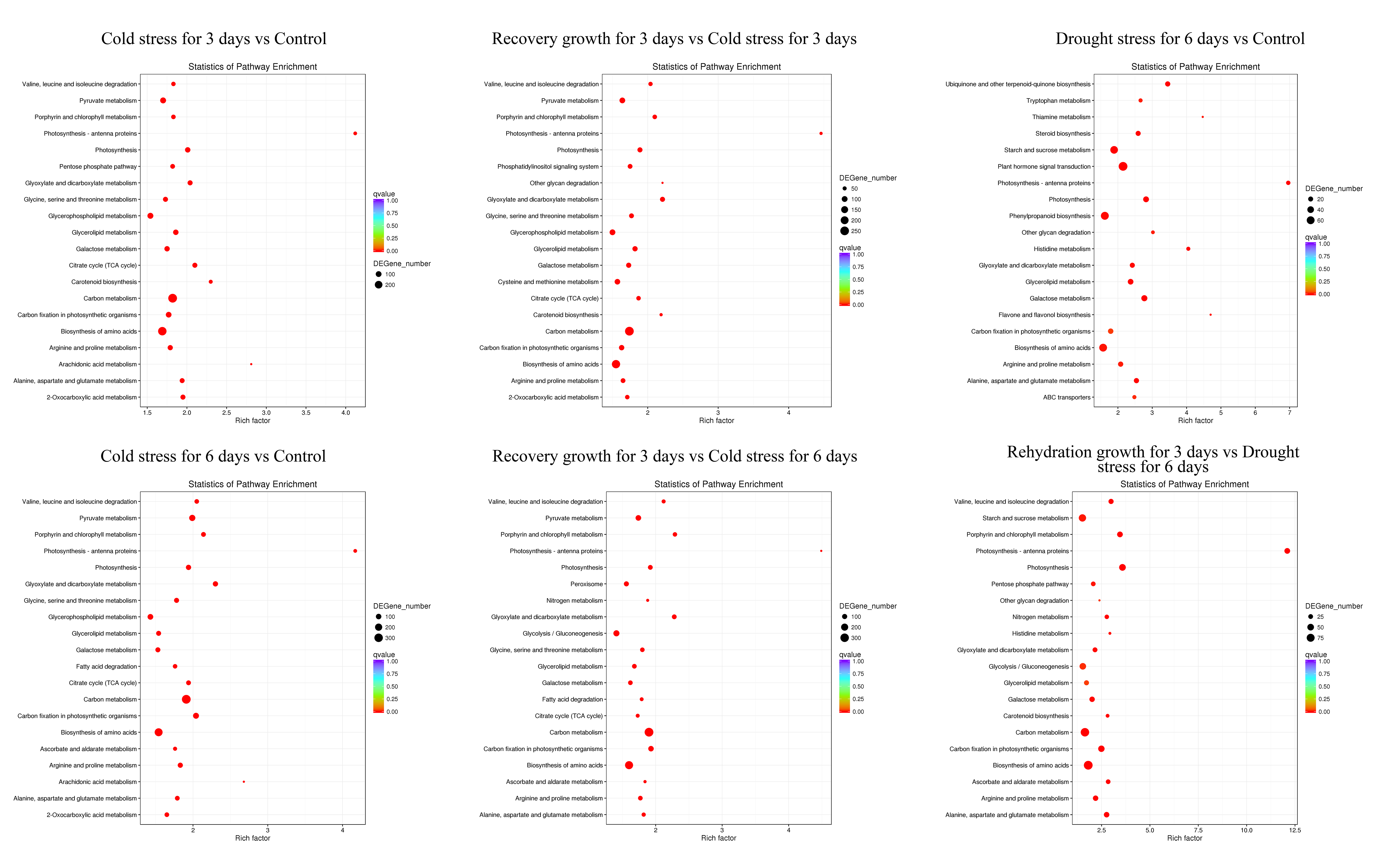

Supplement: Supplementary Figure S11 — KEGG pathway enrichment annotation of DEGs in S. spontaneum in response to cold and drought treatments. [file Image_11.tif]

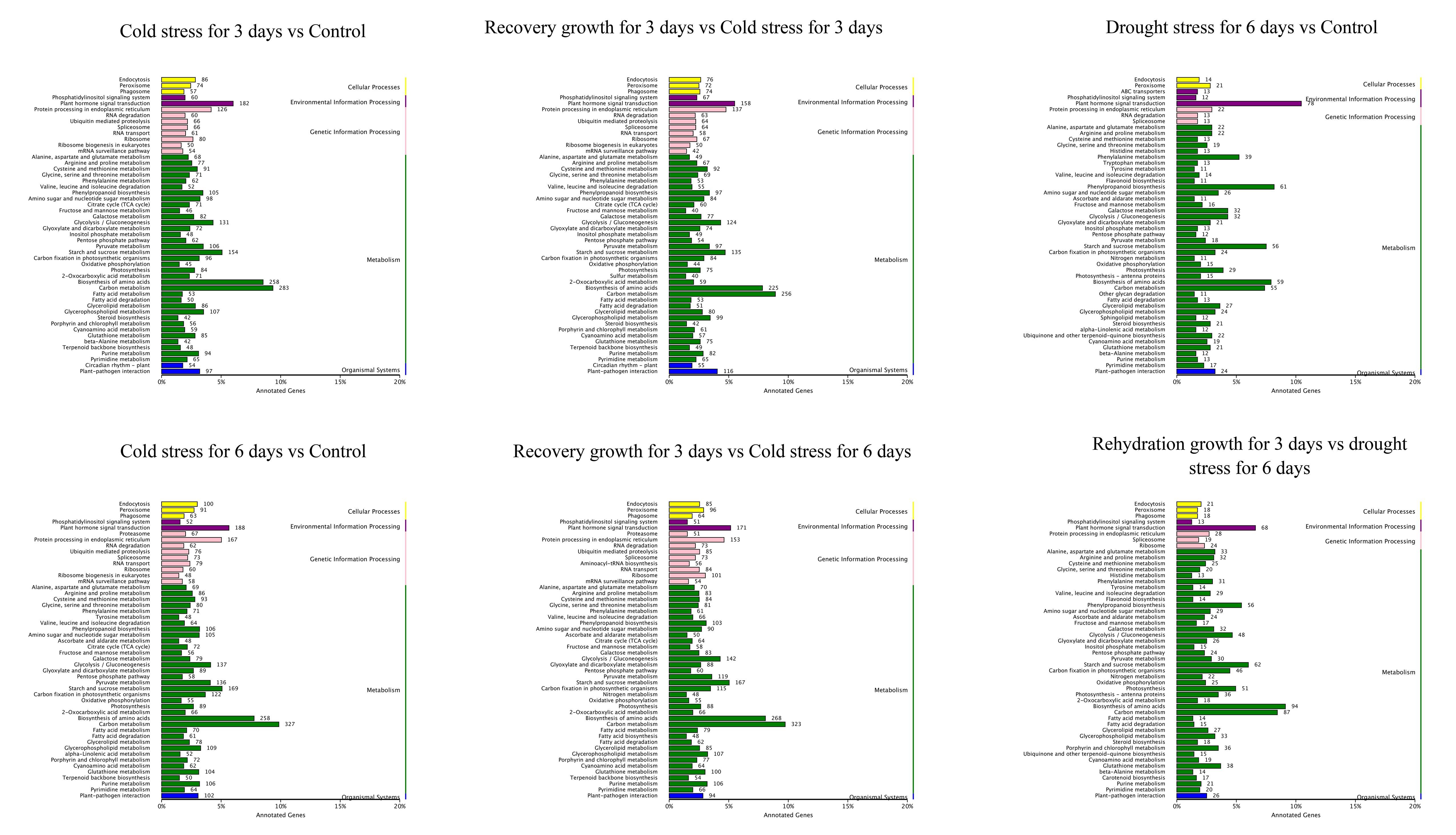

Supplement: Supplementary Figure S12 — The most enriched functional groups under KEGG pathway enrichment annotation for DEGs in S. spontaneum in response to cold and drought treatments. [file Image_12.tif]
